# Smooth Muscle Cell Genome Browser: Enabling the Identification of Novel Serum Response Factor Target Genes

**DOI:** 10.1371/journal.pone.0133751

**Published:** 2015-08-04

**Authors:** Moon Young Lee, Chanjae Park, Robyn M. Berent, Paul J. Park, Robert Fuchs, Hannah Syn, Albert Chin, Jared Townsend, Craig C. Benson, Doug Redelman, Tsai-wei Shen, Jong Kun Park, Joseph M. Miano, Kenton M. Sanders, Seungil Ro

**Affiliations:** 1 Department of Physiology and Cell Biology, University of Nevada School of Medicine, Reno, Nevada, United States of America; 2 Department of Physiology, Wonkwang Digestive Disease Research Institute and Institute of Wonkwang Medical Science, School of Medicine, Wonkwang University, Iksan, Jeollabuk-do, Korea; 3 Aab Cardiovascular Research Institute, University of Rochester School of Medicine and Dentistry, Rochester, New York, United States of America; 4 LC Sciences, 2575 West Bellfort Street Suite 270, Houston, Texas, United States of America; 5 Division of Biological Science, Wonkwang University, Iksan, Jeollabuk-do, South Korea; Georgia Regents University, UNITED STATES

## Abstract

Genome-scale expression data on the absolute numbers of gene isoforms offers essential clues in cellular functions and biological processes. Smooth muscle cells (SMCs) perform a unique contractile function through expression of specific genes controlled by serum response factor (SRF), a transcription factor that binds to DNA sites known as the CArG boxes. To identify SRF-regulated genes specifically expressed in SMCs, we isolated SMC populations from mouse small intestine and colon, obtained their transcriptomes, and constructed an interactive SMC genome and CArGome browser. To our knowledge, this is the first online resource that provides a comprehensive library of all genetic transcripts expressed in primary SMCs. The browser also serves as the first genome-wide map of SRF binding sites. The browser analysis revealed novel SMC-specific transcriptional variants and SRF target genes, which provided new and unique insights into the cellular and biological functions of the cells in gastrointestinal (GI) physiology. The SRF target genes in SMCs, which were discovered *in silico*, were confirmed by proteomic analysis of SMC-specific *Srf* knockout mice. Our genome browser offers a new perspective into the alternative expression of genes in the context of SRF binding sites in SMCs and provides a valuable reference for future functional studies.

## Introduction

Smooth muscle cells (SMCs) possess phenotypic plasticity, which enables them to dedifferentiate and proliferate inappropriately under pathological conditions [1,2]. This phenotypic transition involves genetic reprograming that results in suppression of smooth muscle (SM) contractile gene expression and induction of synthetic genes that are active during hyperplasia and hypertrophy [3]. Over the past few decades, our knowledge about the phenotypic changes of dedifferentiated SMCs that result from SM injury has advanced significantly. This advancement includes the identification of many SMC-specific proteins that are lost during a phenotypic switch [4]. However, the study of SMCs upon injury has been limited by the lack of a comprehensive reference of genome-wide transcripts (transcriptome) from differentiated SMCs.

The contractile function of SMCs is linked to changes in intracellular ion concentrations, which are regulated by ion channels and transporters [5]. Several molecular mechanisms of SMC contraction triggered by these ion channels have been proposed for different SM-based organs [6]. In the GI SM, excitation–contraction coupling occurs by Ca^2+^ entry via voltage-dependent Ca^2+^ channels and Ca^2+^ release from the sarcoplasmic reticulum [7]. A few of the ion channels expressed in SMCs have been discovered. However, to uncover the molecular and cellular mechanisms involved in SMC contraction, identification of all ion channels and transporters expressed in SMCs is required.

The genes responsible for SMC contractility are regulated by serum response factor (SRF). This transcription factor activates gene transcription by binding to a consensus sequence (CC [A/T]_6_ GG) referred to as “CArG box,” which is found in the promoter or intronic regions of many SM-restricted genes [8]. Several functional CArG boxes have been identified in the genome [9]. However, the functional nature of CArG box associated genes in the SMC genome (collectively referred to as the “CArGome”) remains unknown. Since SRF initiates transcription by binding to CArG boxes, identification and analysis of the SMC CArGome would enable the discovery of new SRF-targeted genes, whose expression may be altered in phenotypically changed SMCs.

In addition, several SM-restricted genes, such as myocardin, have been reported to be expressed as splice variants associated with alternative functions in SMCs [10]. Although implicated in the contractile phenotypic diversity of vascular SM, very little is known about exact significance of these alternatively spliced and/or differentially initiated transcriptional variants of SM genes [11]. Therefore, the identification of all transcriptional variants in SMCs is highly desirable to understand their functional significance and to enable analysis of gene expression and regulation of each transcriptional variant. Furthermore, the transcriptional variant sequences could predict the amino acid sequences, which can offer critical clues to the potential functions of the protein products.

Previously, our laboratory developed a method to isolate SMCs using transgenic mice that ectopically express enhanced green fluorescent protein (eGFP) [12]. Using this eGFP-based separation method, we were able to study downstream gene expression and determine the specific functional roles of the cell type.

Here we report the complete transcriptomes of SMCs derived from the mouse jejunum and colon. We chose the jejunum and colon SMCs for this project because these distinct parts of the intestine have different electrophysiological and pharmacological characteristics. For example, the colon has a motor pattern that is different than that of the small intestine, which results in a slower transit time in the colon. Identification of differentially started or spliced genes in the respective transcriptomes could potentially explain the functional differences between the two SMs. We also report an analysis of the 16,000 genes found in the transcriptome, which led to the discovery of 55,000 transcriptional variants. This includes the identification of several hundred ion channels and transporters as well as SMC-specific genes that are characteristic of its cellular identity and function. The transcriptome information was imported into a custom-built SMC genome browser, which interacts with the publically available genome bioinformatics data in the University of California, Santa Cruz (UCSC) genome database [13]. The genome browser serves as a reference that provides important information regarding the possible structure, isoforms, and regulation of expression of all genes expressed in SMCs. Furthermore, the browser also enables identification of SRF binding sites that may be involved in the transcriptional initiation of SM-restricted genes.

## Materials and Methods

### Animal and tissue preparation

Jejunum and colon *tunica muscularis* were obtained from *smMHC* ^*Cre-eGFP/+*^ mice [14]. These tissues were then used to isolate SMCs. Jejunum was also obtained from inducible SMC-specific *Srf* knockout (*smMHC* ^*Cre-ERT2/+*^ *;Srf* ^*lox/lox*^) mice [15,16] injected with tamoxifen (1 mg/100 μl) for five consecutive days. Animal protocol was approved by the Institutional Animal Care and Use Committee at the University of Nevada.

### Flow cytometry and fluorescence-activated cell sorting (FACS)

Cells were dispersed from jejunum and colon muscularis, and eGFP^+^ SMCs were sorted from dispersed cells using FACS as described [12]. Isolated SMCs were pooled from approximately 40 mice and used to isolate total RNAs as one collective sample.

### Isolation of total RNAs

Total RNAs were isolated from jejunum muscularis, colon muscularis, jejunal smooth muscle cells (JSMCs), and colonic smooth muscle cells (CSMCs) using the mirVana miRNA isolation kit (Life Technologies, Carlsbad, CA, USA). Quality of total RNAs was analyzed via NanoDrop 2000 Spectrometer (Thermo Scientific, Waltham, MA, USA) and 2100 Bioanalyzer (Agilent Technologies, Santa Clara, CA, USA).

### RT-PCR

The cDNA libraries were transcriptionally constructed from total RNAs isolated from jejunal and colonic SM tissues as well as from FACS purified SMCs as previously described [12]. PCR analysis on cDNAs was also performed as previously described [12]. All primers used for RT-PCR are shown in S11 Table.

### Construction of RNA-seq libraries and next-generation sequencing

Four RNA-seq libraries were generated using Illumina’s sample preparation Kit according to the manufacturer’s instructions. The cDNA libraries were sequenced via Illumina HiSeq 2000 (Illumina, San Diego, CA, USA) following the vendor’s instruction at LC Sciences (Houston, TX, USA).

### Bioinformatics data analysis

Paired-end sequencing reads were aligned against the reference genome (UCSC mm9) using TopHat v1.4.1 software. The assembly was conducted, and the expression level was estimated in fragments per kilobase of transcript per million fragments mapped (FPKM) using Cufflinks v2.0.2 software. The Cufflinks software estimated the FPKM value and their 95% confidence intervals. Transcripts with a lower confidence interval boundary of FPKM equal to 0 was considered as “unreliable” and transcripts with a lower confidence interval boundary of FPKM larger than 0 was considered as “reliable.” Reliability was plotted against the logarithm of the FPKM value to determine the cutoff. A cutoff of FPKM equal to 0.025 generated equal false positive and false negative ratios of reliability. The expression levels of transcripts with FPKM values less than 0.025 was considered to be 0.

### Mouse CArGome

A custom Perl script was created to identify CArG elements genome-wide (CArGome) in *mus muscularis*. The specific regular expressions matched are listed in S10 Table. Conserved CArG boxes were identified via comparison between mouse CArGome and human CArGome [17]. Mouse and conserved CArG boxes were documented in the BEB format.

### Confocal microscopy and immunohistological analysis

Microscopic analysis of eGFP fluorescence in the jejunum and colon was performed as described with minor modifications [18]. For whole mount immunohistochemistry, tissues were fixed in 4% paraformaldehyde in PBS and were stained with anti-MYH11 primary antibody (1:300 dilution, Alfa Aesar, Ward Hill, MA, USA) and Alexa Fluor 594 Donkey anti-Rabbit IgG secondary antibody (1:1000 dilution, Life Technologies, Carlsbad, CA, USA). Images were collected using the Fluoview FV10-ASW 3.1 Viewer software (Olympus, Tokyo, Japan) with an Olympus FV1000 confocal laser scanning microscope.

### Western blot

Protein was extracted from tissue samples from *Srf* knockout (KO) and wild type (WT) mice and Western blotting was performed as previously described [19]. Primary antibodies against the following antigens were used: RYR3 (rabbit, 1:1200, Alomone Labs, Jerusalem, Israel), SRF (rabbit, 1:500, Santa Cruz Biotechnology, Dallas, TX, USA), or GAPDH (mouse, 1:1500, Abcam, Cambridge, MA, USA).

### Proteomics analysis

Soluble proteins were prepared from jejunum of WT and *Srf* KO mice. Proteins in the extracts were trypsin digested and analyzed by two-dimensional LC-MS/MS on a Thermo Finnigan LTQ-Orbitrap (Thermo Scientific, Waltham, MA, USA). Data were analyzed using Sequest and validated with Scaffold proteome software (Proteome Software, Portland, OR, USA). Proteins were quantified by spectral counting, and potentially perturbed molecular pathways were identified using Ingenuity Computational Pathway Analysis (Ingenuity, Redwood City, CA, USA). Differentially expressed proteins in *Srf* KO SMCs were obtained and analyzed with SRF-associated genes.

### Availability of supporting data

SMC transcriptome and CArGome data were deposited in the custom track of the UCSC genome database [13]. UCSC Smooth Muscle Genome Browser is available at http://medicine.nevada.edu/physio/transcriptome (requires Google Chrome and takes ~1 minutes to upload the large files). The genome browser contains the transcriptome menus on the “Custom Tracks.” Each menu has different display options.

The abbreviated instructions are as follows: 1) To search transcriptional variants of a gene, type in the gene symbol, and click “go.” 2) Under “Custom Tracks,” select the view option (e.g., “full”) for type of sample (e.g., “SMC_Jejunum”), and click “refresh.” 3) Select the bioinformatics data of interest (e.g., click on “full” under “RefSeq Genes” in “Genes and Gene Predictions” and/or “Caltech TFBS” under “Expression and Regulation” for SRF binding sites), and then click “refresh.” 4) Select options in each bioinformatics data (e.g., click “Caltech TFBS,” select “SRF,” change the display mode to “full,” and click “Submit”). 5) Click “configure” to optimize views (change image width and text size).

The RNA-seq data from this study have been also submitted to the NCBI: GSM1388412, SM Jejunum; GSM1388413, SM Colon; GSM1388406, SMC Jejunum; GSM1388407, SMC Colon.

## Results

### Identification and isolation of differentiated SMCs

**Fig 1:**
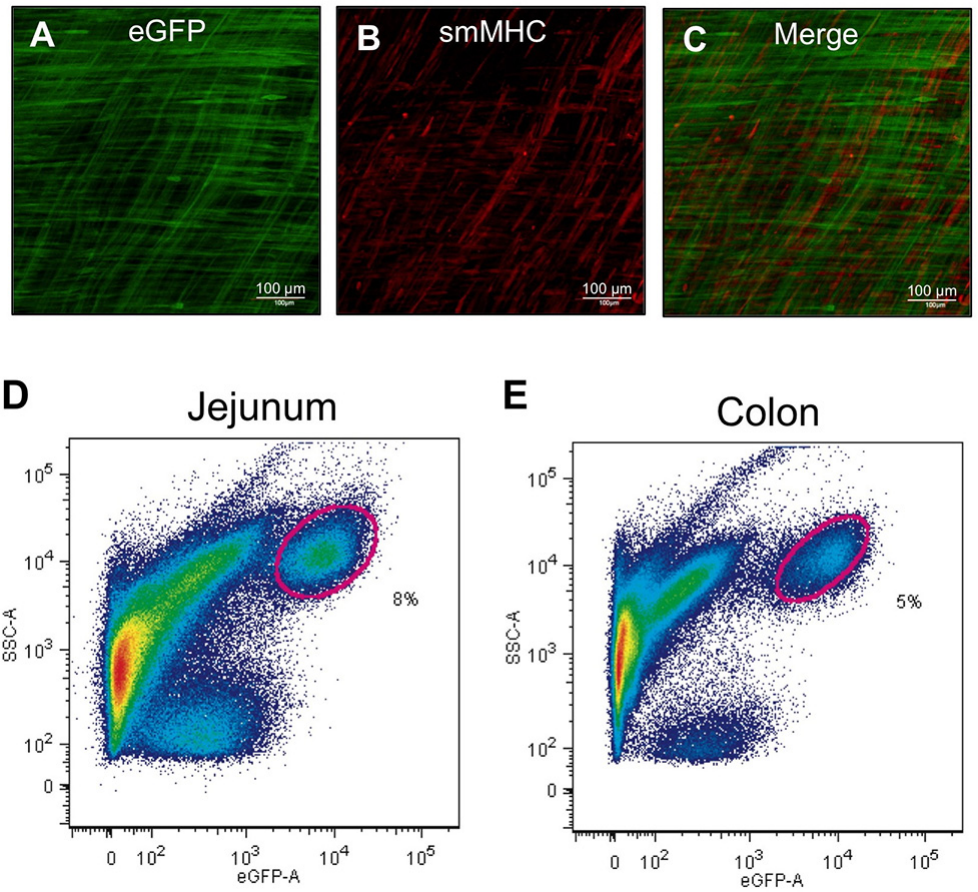
Identification of SMCs in the intestinal smooth muscle with eGFP and MYH11 antibody. (A) A confocal microscopy z-stack image of whole-mount jejunum muscularis showing longitudinal and circular SMCs expressing eGFP. (B) Immunohistochemistry of SMCs using anti-MYH11(smMHC) antibody. (C) Merged images of eGFP and MYH11(smMHC). (D & E) Primary eGFP^+^ SMCs from jejunum and colon identified (circled) on flow cytometry.

Differentiated SMCs were identified in the GI SM of *smMHC* ^*Cre-eGFP/+*^ mice [14] through detection of eGFP-labeled SMCs by confocal microscopy. The phenotypic identity of mature eGFP-labeled SMCs was confirmed by immunohistochemical staining with anti-smMHC (MYH11) antibody, the most selective marker for differentiated SMCs (Fig 1). SMCs in the circular and longitudinal layers of jejunum were labeled by both eGFP and anti-smMHC (MYH11) antibody (Fig 1A–1C). Using cell-specific markers, we previously demonstrated that the eGFP-labeled cells from *smMHC* ^*Cre-eGFP/+*^ were differentiated SMCs [12]. SMCs from the jejunum and colon were likewise analyzed by flow cytometry, through which distinct populations of eGFP^+^ SMCs were identified (Fig 1D and 1E). We sorted the SMCs using FACS for 40 mice and pooled the sorted samples together to isolate the mRNAs for a single RNA-seq analysis.

### Comparison and analysis of SMC transcriptomes in jejunum and colon

**Fig 2:**
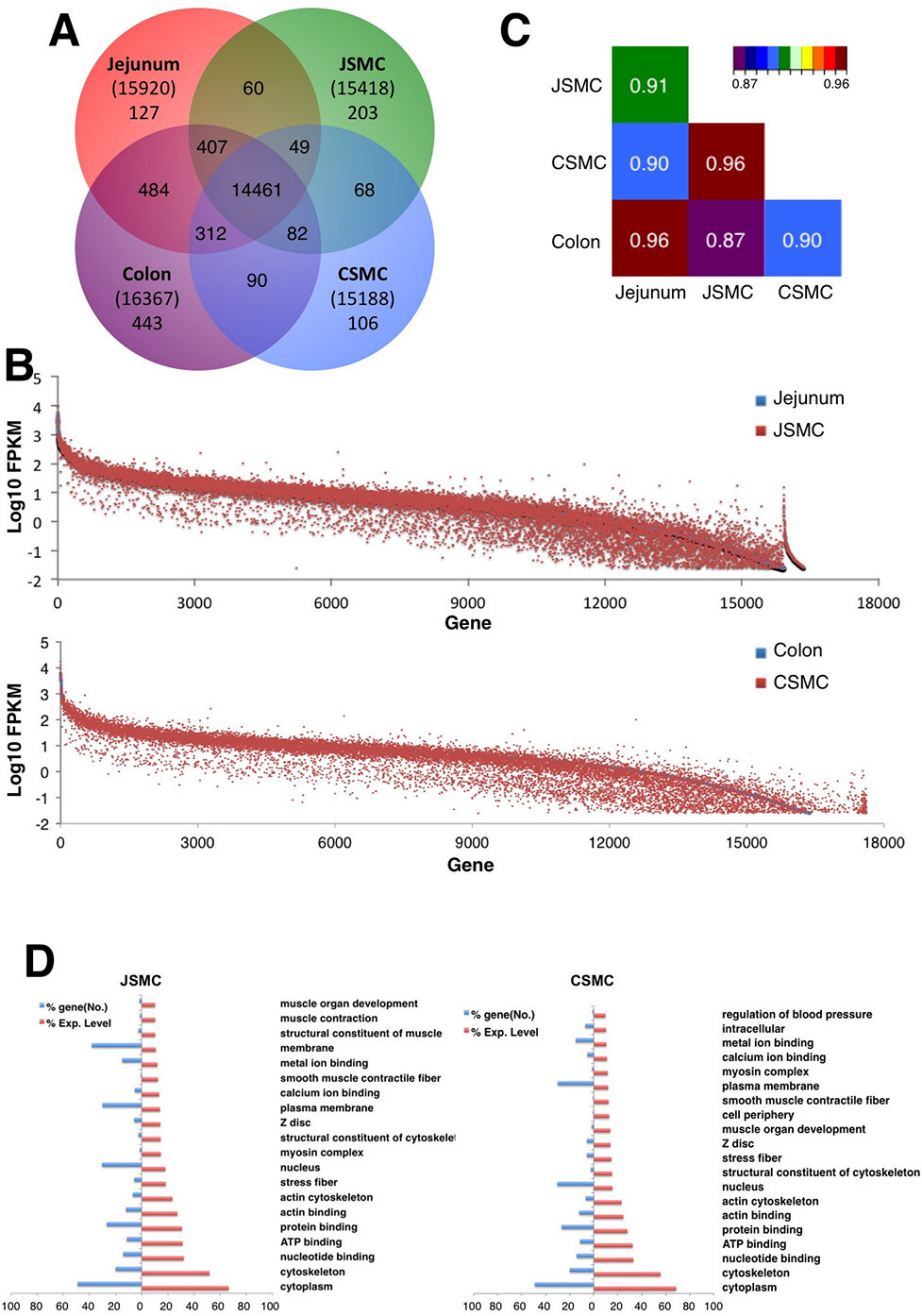
Comparison of transcriptomes obtained from jejunal and colonic SMCs. (A) Venn diagram showing the number of genes expressed in JSMCs, CSMCs, jejununal muscularis, and colonic muscularis. (B) Comparison of expression levels of genes in JSMCs and CSMCs. (C) Comparison of correlation coefficients between JSMCs, CSMCs, jejununal muscularis, and colonic muscularis. (D) Gene ontologies reported in JSMCs and CSMCs. Gene ontology (GO: function, process, and component) of SMC-specific genes was analyzed, and key GO terms were compared with a normalized expression percentile.

To identify all genes expressed in SMCs of jejunum and colon, RNA-seq was performed on mRNA samples from jejunal SM tissue, sorted jejunal SMCs, colonic SM tissue, and sorted colonic SMCs. The transcriptomes included approximately 15,000 to 16,000 known genes (S1 Table). We obtained 151 to 238 million reads, of which 77–93% were mapped onto the genome. Approximately 46,000–55,000 gene isoforms were identified by unique gene annotations. Complete lists of all isoforms identified in this study are shown along with the tracking ID, gene ID and name, chromosome location, isoform length, and expression levels in both jejunal (S2 Table) and colonic SMCs (S3 Table). SMCs expressed an average of three isoforms per gene, which were the result of different transcriptional start sites and/or post-transcriptional alternative splicing (S1 Table). Most genes (14,461) were expressed in both jejunal and colonic tissue, as well as in isolated SMCs, and only a few hundred genes were determined to be cell-specific (non-overlapping single colors in Fig 2A). Complete lists of all of the genes expressed in the jejunal and the colonic SMCs (JSMC and CSMC, respectively) along with their combined expression levels from all of the transcriptional variants are shown in S4 and S5 Tables.

The levels of all genes expressed in SMCs were compared. Several hundred genes were expressed at high levels in both jejunal and colonic SMCs, and the genes that were expressed at low levels had generally a higher discrepancy in their levels between whole muscularis tissues (from jejunum and colon) and their isolated SMCs (Fig 2B). The overall expression profiles between the SMCs from the jejunum and the colon were similar with a correlation coefficient of 0.96 (Fig 2C). Correlation coefficients between the SMCs and the tissue type were also similar with a value of 0.91 (jejunal SMCs and tissue) and 0.90 (colonic SMCs and tissue). Since the expression profiles of SMCs and tissues were similar, we examined the cellular markers of GI tissue [*Myh11* for SMCs, *Kit* for ICC, *Pdgfra* for PDGFRα^+^ cells, and *Uchl1* (known as PGP9.5) for neuronal cells] in order to validate the identity of the cells [7]. Each cellular marker was expressed abundantly in both jejunal and colonic tissues (S1A–S1D Fig). However, as predicted, only *Myh11* was highly expressed in jejunal and colonic SMCs (S1A Fig). Expression of *Myh11* in jejunal and colonic SMCs was significantly higher than in the corresponding jejunal and colonic muscularis tissue.

An investigation of the SMC gene ontology (GO), which was derived from the transcriptome, revealed key GO terms that are related to the physiologic function of SMCs (Fig 2D). Most of the GO terms were shared by jejunal and colonic SMCs and included cytoskeleton, actin binding, calcium ion binding, myosin complexes, and SM contractile fibers, which are involved in muscle contraction (Fig 2D). The similarity of the GO terms between SMCs from jejunum and colon suggested that these two groups of cells have similar muscle functions even though they are located in different regions of the GI tract.

### Construction of an SMC genome browser

**Fig 3:**
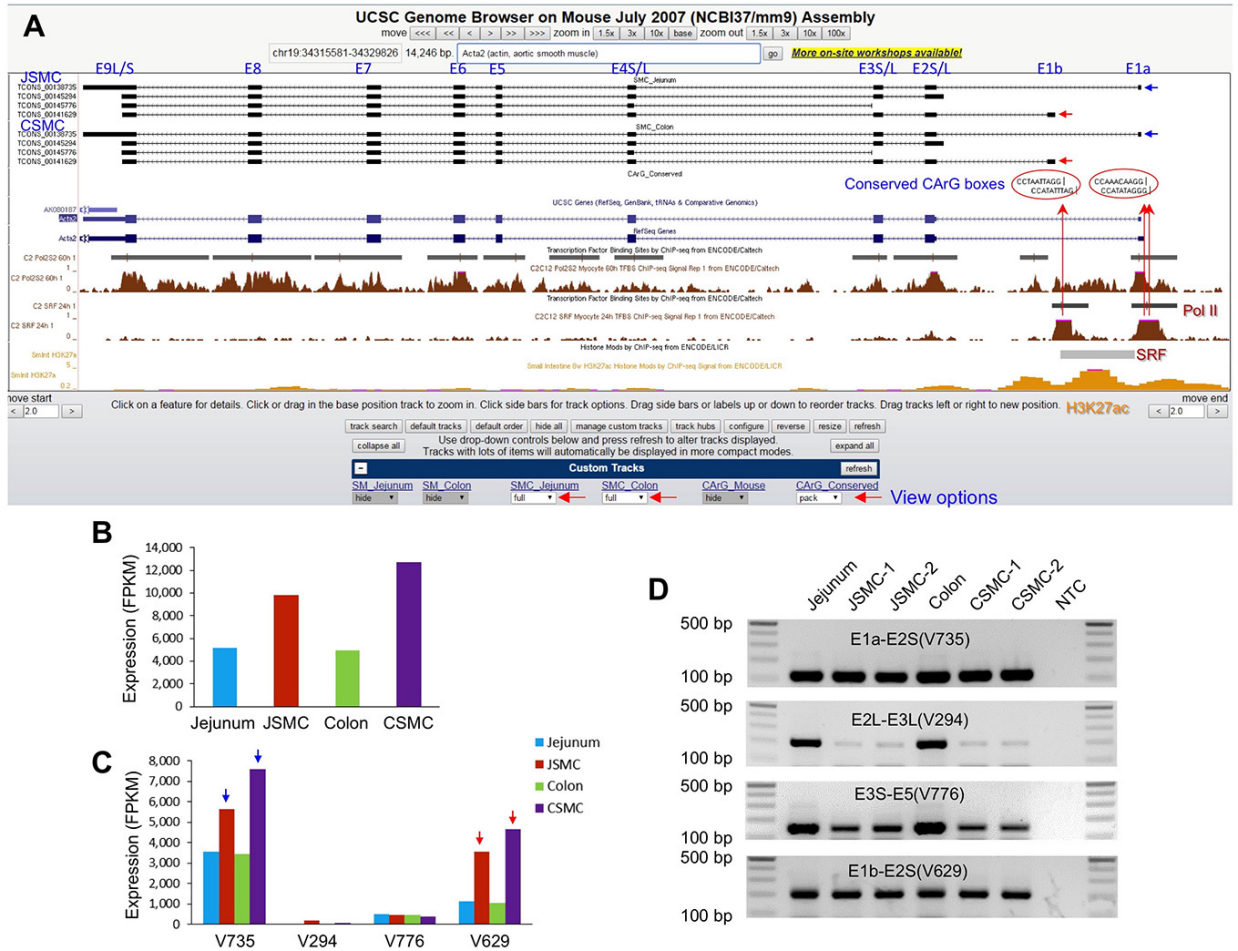
SMC transcriptome, SRF binding sites, and CArG boxes built on the UCSC genome browser. (A) A genomic map view of CArG boxes and SRF binding sites on *Acta2*, a gene expressed in SMCs. All predicted CArG boxes were mapped on the genome. Conserved CArG boxes beween mice and humans are indicated by red circles. Four transcriptional variants with alternative initiation sites were expressed in colonic and jejunal SMCs (set by view options; large red horizontal arrows). The transcriptional variants were aligned with H3K27ac sites (small intestine), RNA polymerase II and SRF binding sites (C2C12 myoblasts), which were publically available in the UCSC database. The two SRF binding sites contained three CArG boxes (red circles), which were located in the promoter and intron 1 regions, and conserved beween mice and humans (set by view options). Note that the SRF binding sites aligned with that of RNA polymerase II and the H3K27ac sites from SRF ChIP data. Directionality of each mRNA on the browser is indicated by the arrows on a line (e.g., >>>, sense strand; <<<, antisense strand). (B) Expression levels (FPKM) of *Acta2* in SMCs and whole tissue. (C) Expression levels (FPKM) of *Acta2* transcriptional variants in SMCs. The variant (V) ID represents the last three digits of the TCONS number. (D) PCR validation of *Acta2* exons with different transcriptional initiation sites in isolated SMCs and muscularis of jejunum and colon. NTC is non-template control. Primer sets were designed from variant exons in the regions of interest (see S11 Table for primer sequences).

An interactive SMC genome browser was created in order to analyze the SMC-specific genes using the UCSC genome database [13]. This genome browser provided the genomic structure of each transcriptional variant, including promoter regions, exons, and introns, for all genes expressed in SMCs (Fig 3A). The browser also enabled analysis of our transcriptome data using the gene expression and regulation data that have been compiled into the UCSC genome database by other researchers. Since the expression of SMC contractile genes is regulated by SRF recognition and binding of CArG boxes within the promoter regions of its target genes [8], a genome-wide search for CArG boxes (“CArGome”) was conducted. Approximately 3,106,586 CArG boxes were found in the mouse genome, and many SRF-binding CArG boxes were conserved between humans and mice [9]. This prompted a search of the human genome, which resulted in identification of 98,236 CArG boxes that were conserved between humans and mice. A search of the SMC transcriptome and the CArG boxes was performed in order to identify CArG boxes that are involved in regulation of transcription. The mouse CArGome was uploaded to the UCSC database genome browser containing the previously mapped genome-wide SRF binding sites of the mouse muscle myoblast cell line (C2C12) [13]. Although C2C12 cells were derived from mouse thigh muscle, SRF regulates cytoskeletal genes through the same CArG boxes in the cells as in SMCs and therefore, share a common pathway of gene regulation [20].

CArG boxes and SRF binding sites were analyzed using the genome browser because *Acta2* (SM actin alpha 2), which is expressed in vascular SMCs, is regulated by SRF [21] and abundantly expressed in both jejunal and colonic SMCs (Fig 3B, S4 and S5 Tables). There were four expressed transcriptional variants of *Acta2* that contained alternative transcriptional initiation sites in both JSMCs and CSMCs (Fig 3A). The two main *Acta2* variants highly expressed in SMCs were TCONS_00138735 (1,780 bp) and TCONS_00141629 (1,313 bp; Fig 3C, S2 and S3 Tables). These two variants, which start on E1a (TCONS_00138735) and E1b (TCONS_00141629), were also found in humans (NM_001613) as well as in other tissues in mice, such as the prostate (DV047329). In addition, an *Acta2* variant starting on E2L (TCONS_00145294) was also found in humans (NM_001141945). The expression of all four *Acta2* variants in JSMCs, CSMCs, jejunum, and colon tissue was validated by RT-PCR (Fig 3D). As anticipated, the promoter regions of the two main variants contained SRF binding sites (Fig 3A). These two binding sites contained the conserved CArG box sequences CCATATAGGG and CCAAACAAGG on the first SRF binding site and CCTAATTAGG on the second SRF binding site. Collectively, the genomic map, SRF binding sites, CArG boxes, and variant expression data of *Acta2* all suggested that SRF binding of the three different CArG boxes drives the expression of the two dominant transcriptional variants in SMCs.

Since SRF is also known to self-regulate the *Srf* gene [22], we also investigated whether SRF binding sites are located within the RNA polymerase II binding site and whether transcriptional initiation of *Srf* variants may also be influenced by neighboring CArG boxes. Our transcriptome data showed that the *Srf* gene encodes seven exons and has three different transcriptional initiation sites located on exon 1 (E1a, E1b, and E1c) resulting in the expression of three variants in JSMCs and CSMCs (S2A Fig). *Srf* was highly expressed in both SMCs (S2B Fig, S4 and S5 Tables), and the two main variants were TCONS_00115860 and TCONS_00126017 starting at E1a and E1b, respectively (S2A and S2C Fig). These two variants started from a CpG island promoter region that overlaps with a RNA polymerase II binding site (C2C12 SRF ChIP-seq from ENCODE/Caltech data; S2A Fig). The polymerase binding region also contained a SRF binding site that aligned with the three CArG boxes CCATATAAAG, CCTTTTATGG, and CCTTATATGG conserved between mice and humans. There was an additional SRF binding site in intron 2 that contained the CArG box CCTTATATGG, which was also conserved between mice and humans. Finally, we validated the expression of all three variants in JSMCs and CSMCs by RT-PCR (S2D Fig). Taken together, the RT-PCR validation of our bioinformatics analysis confirmed that our SMC transcriptome data and the interactive SMC genome browser are accurate and reliable.

### Analysis of CArGome and SRF binding sites in the genes of SMCs

**Fig 4:**
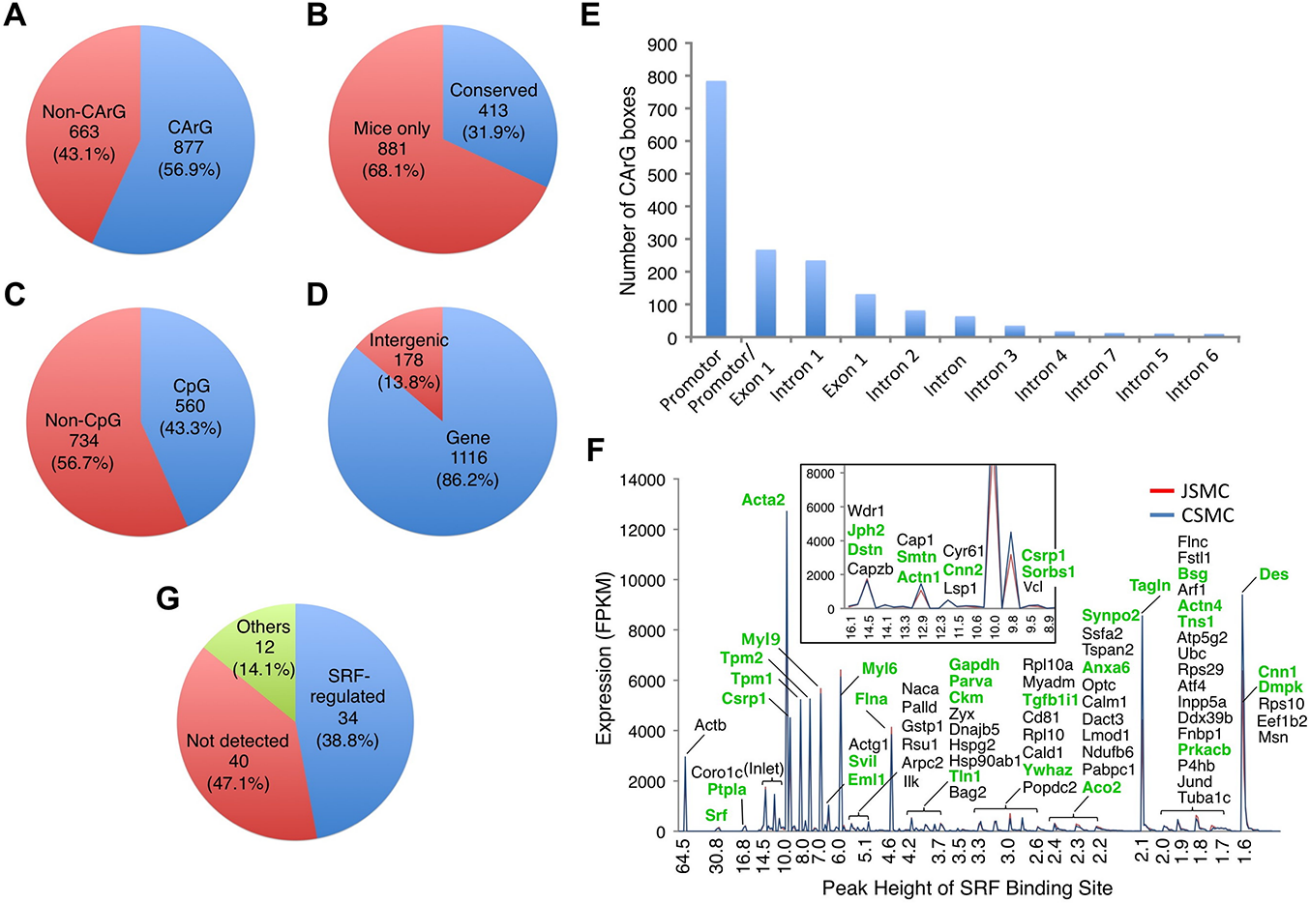
Analysis of CArG boxes and SRF binding sites on SMC transcriptome. A total of 1,540 SRF binding sites (peak height >1.5) obtained from SRF ChIP-seq of C2C12 myoblasts were analyzed for association with CArG boxes, CpG islands, and genes. (A) Proportion of SRF binding sites associated with CArG boxes. (B) Proportion of SRF binding sites associated with CArG boxes conserved between mice and humans. (C) Proportion of SRF binding sites associated with CpG islands. (D) Proportion of SRF binding sites associated with genes. (E) Locations of SRF binding sites on genes expressed in SMCs. (F) A representative list of SMC genes that were highly expressed and associated with SRF binding sites. Eighty-six genes containing SRF binding sites (peak hight >1.5) and highly expressed (>100 FPKM) in SMCs are shown. The SMC genes of the 34 proteins regulated by SRF in SMC-specific *Srf* KO mice are indicated in bold green letters. (G) Proportion of SRF-regulated proteins identified in jejunal SM from SMC-specific *Srf* KO mice. Eight-six SRF-associated genes were compared with the SRF-regulated proteins identified in a proteomics study involving *Srf* KO mice: 34 proteins were down-regulated (SRF-regulated; blue), 40 proteins were not detected (red), and 12 proteins did not change expression levels (others; green) in the *Srf* KO SM.

Since alternative SMC transcripts appeared to be closely associated with binding of SRF to different CArG boxes, we performed an analysis of all SRF binding sites in the mouse genome. A total of 6,759 SRF binding sites were identified in the myocyte genome, and 1,540 of these binding sites were selected for further analysis based on the strength of the SRF signal (peak height > 1.5 signal value). A complete list of SRF binding sites associated with CArG boxes, CpG islands, and JSMC transcripts is shown in S6 Table. Fifty-seven percent (877) of SRF binding sites were associated with CArG boxes, and 32% (413) of the SRF binding sites were conserved between humans and mice (Fig 4A and 4B). Approximately 43% (560) of all SRF binding sites were found within the CpG islands of transcriptional start sites (Fig 4C). Most SRF binding sites were found within the promoter regions and were followed by exon 1 or intron 1 (Fig 4E), and a total of 1,116 genes (86%) were associated with SRF binding sites (Fig 4D), of which most were abundantly expressed in SMCs. There were 86 genes that were closely associated with SRF binding sites and highly expressed in SMCs (Fig 4F). Of the SRF-associated genes, several new genes were identified in additional to known contractile genes such as *Acta2*, *Tagln*, and *Des* (Fig 4F).

In a parallel study, we generated a tamoxifen-induced SMC-specific *Srf* KO mouse line and performed a comprehensive proteomic analysis to detect the quantitative changes of protein expression that occurs with *Srf* deletion in the SM. The proteomic analysis identified 50 proteins that were significantly decreased in the *Srf* KO SM (manuscript in submission). We then compared the 86 SRF-associated genes to the down-regulated proteins identified in *Srf* KO mice (Fig 4F and 4G). A total of 34 down-regulated proteins correlated with the identified SRF-associated genes expressed in SMCs, thereby supporting that they may be direct targets of SRF in SMCs (Fig 4G and S7 Table).

### Identification of SMC-specific genes

**Fig 5:**
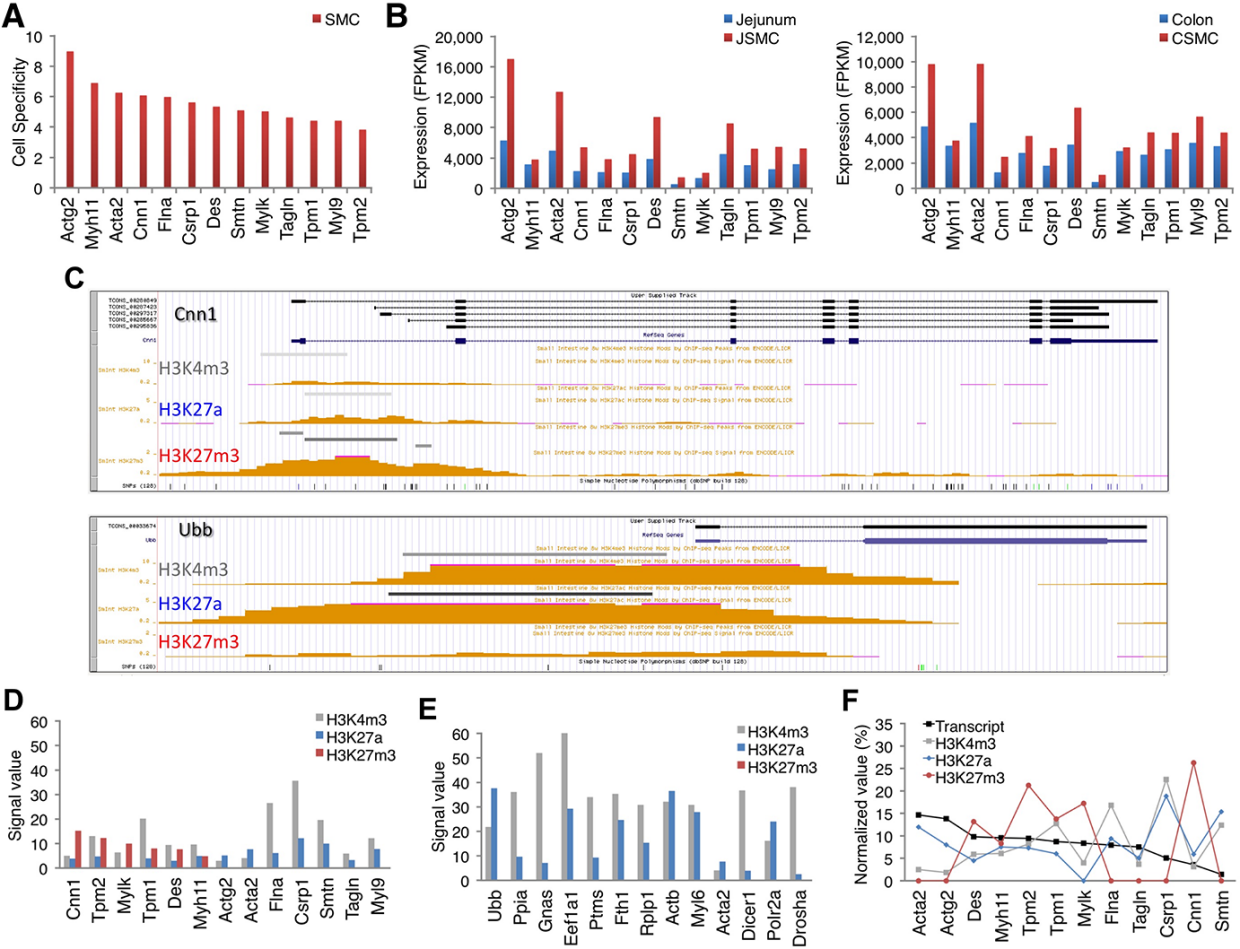
Identification of SMC-specific genes by expression profiles and histone modifications. (A) Specificity of SMC-enriched genes. Cell specificity was determined by comparative analysis of gene expression profiles among SMCs, ICCs, and PDGFRα^+^ cells: SMCs^expression level (FPKM)^/[ICC^expression level (FPKM)^ + PDGFRα^+^ cells^expression level (FPKM)^] (B) Comparison of JSMC- and CSMC-enriched gene expression. (C) Genomic map views of histone modications (H3K4m3, H3K27a, and H3K27m3 in 8 week small intestine) on *Cnn1* (specifically expressed in JSMCs and CSMCs) and *Ubb* (ubiquitiously expressed). (D) Comparison of histone modifications on SMC-enriched genes. The signal value is the average of the mininum and maxium values of the ChIP-seq signal for each histone modification. (E) Comparison of histone modifications on ubiquitious genes. (F) Expression levels of SMC-enriched genes not altered by histone modifications.

In order to identify distinctive cellular markers for SMCs, genes preferentially expressed in SMCs were analyzed. In parallel studies, we also obtained the transcriptome data from interstitial cells of Cajal (ICC) and PDGFRα^+^ cells of mouse jejunum and colon (manuscript in submission). Comparative analysis of gene expression profiles among SMCs, ICC, and PDGFRα^+^ cells identified cell-specific genes. These genes included known markers of SMC differentiation such as *Actg2*, *Myh11*, *Acta2*, *Cnn1*, *Des*, *Tagln*, and *Smtn* as well as several genes that had not been previously associated with GI SMCs. The novel genetic markers identified included *Flna*, *Csrp1*, *Mylk*, *Tpm1*, *Myl9*, and *Tpm2* (Fig 5A). We found that *Actg2*, *Myh11*, *Acta2*, and *Cnn1* were the most cell specific genetic markers in both JSMCs and CSMCs (Fig 5B).

Recent studies have shown unique histone methylation and acetylation markers for gene expression in human and mouse cells. These include H3K4m3 for active or poised genes; H3K27a for active genes; and H3K27m3 for silenced genes [23–25]. Since gene expression in SMCs is also regulated in part by histone modifications [26], we reanalyzed the SMC-specific genes along with the ubiquitously expressed genes via the genome browser, which incorporated the small intestine histone modification maps available at the public mouse encyclopedia of DNA elements (mouse ENCODE) [27]. Interestingly, SMC-specific genes had histone modifications that were unique compared to ubiquitously expressed genes. For example, our RNA-seq data showed that the *Cnn1* mRNAs, which were specific to SMCs, were enriched in both JSMCs and CSMCs (S3B Fig). This gene was expressed as five variants in SMCs (S2 and S3 Tables), which had different transcriptional start sites in exon 1 (Fig 5C and S3A Fig). Two main *Cnn1* transcripts in the SMCs were TCONS_00280849 and TCONS_00295836 (S3C Fig). Expression of all five variants of the gene were validated in both sources of SMCs by RT-PCR (S3D Fig). The transcriptional start sites of *Cnn1* contained low levels of H3K4m3 and H3K27a but higher levels of H3K27m3 suggesting that the gene is active in SMCs and inactive in non-SMCs within the small intestine (Fig 5C). In contrast, *Ubb* was expressed as a single variant, and its promoter region had high levels of H3K4m3 and H3K27a modifications but very low levels of H3K27m3 suggesting that the gene is active in all cells within the tissue rather than just in SMCs (Fig 5C). These histone modification signatures allowed for the identification of six SMC-specific genes in the transcriptome. The six genes include three well-known markers *Cnn1*, *Myh11*, and *Des* and three new markers *Tpm1*, *Tpm2*, and *Mylk*, which are specific to primary SMCs (Fig 5D). Further testing of the twelve most ubiquitously expressed genes in SM tissue and cells summarily confirmed the presence of the expected histone modification signature (Fig 5E). However, the expression levels of SMC-specific genes appeared to be unrelated to the degree of histone modification suggesting that other transcriptional regulators may control these genes (Fig 5F). Furthermore, we confirmed that six of the SMC-specific genes contained multiple conserved CArG boxes in the promoter or intronic regions and that three of the genes (*Tpm1*, *Tpm2*, and *Cnn1*) contained conserved CArG boxes, which aligned well with the publicly available data (C2C12) on SRF binding sites (S4A–S4C Fig). The SRF binding sites for the other three genes (*Mylk*, *Des*, and *Myh11*) were unfortunately unavailable in the public database indicating that they may be expressed in minute levels in C2C12 myocytes. Taken together, the genomic analyses showed that SMC-specific genes may be regulated by SRF and histone modifications.

### Comparative analysis of ion channels and transporters expressed in SMCs

**Fig 6:**
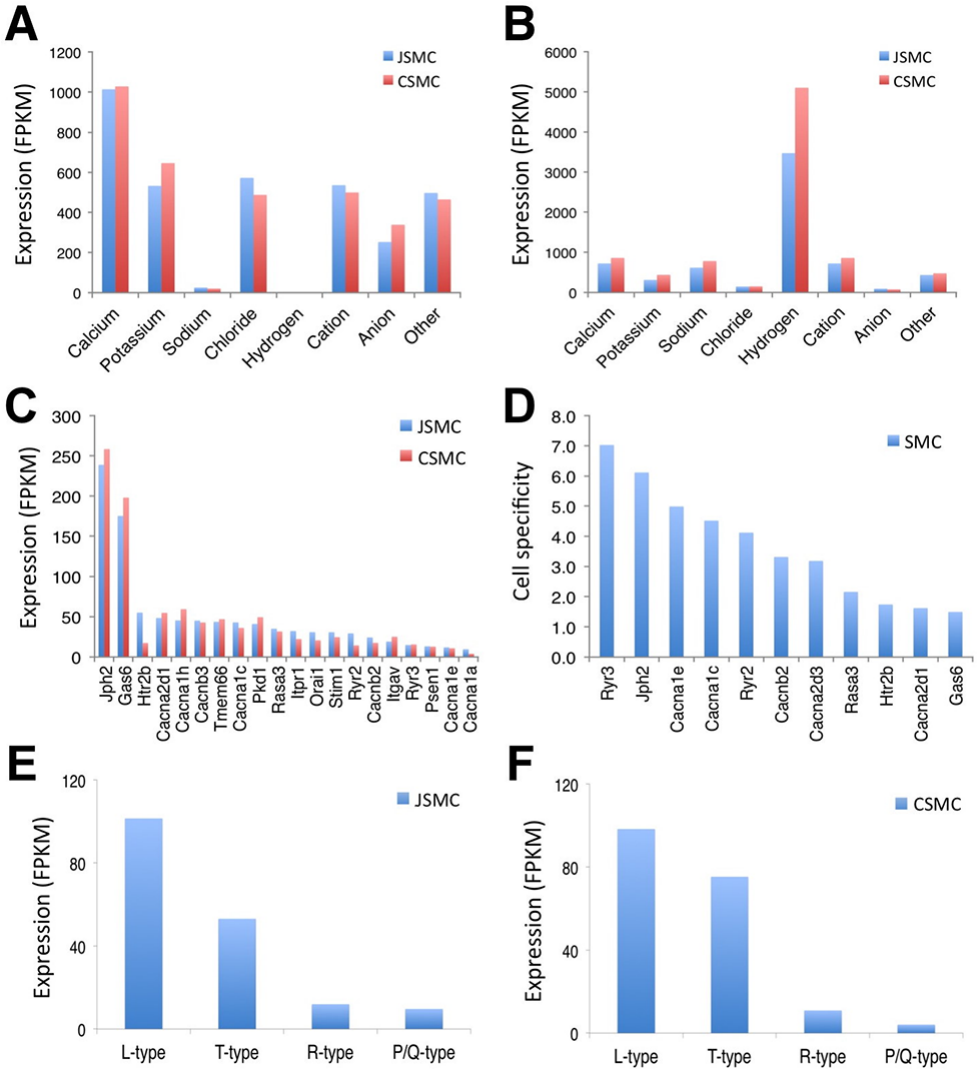
Comparison of ion channel and transporter isoform genes expressed in SMCs. (A) Comparison of expression levels of ion channel isoforms in JSMCs and CSMCs. (B) Comparison of expression levels of ion transproter isoforms in JSMCs and CSMCs. (C) Calcium channel isoforms enriched in JSMCs and CSMCs. (D) SMC-specific calcium channel isoforms. Cell specificity was determined by comparative analysis of gene expression profiles among SMCs, ICC, and PDGFRα^+^ cells. (E) Voltage-dependent calcium channel isoforms (L-type, *Cacna1c* & *d*; T-type, *Cacna1h* & *g*; R-type, *Cacna1e*; P/Q-type, *Cacna11*) expressed in JSMCs. (F) Voltage-dedendent calcium channel isoforms in CSMCs.

Since contractile activity of SMCs is regulated by Ca^2+^ via ion channels and transporters [7,28], we used the transcriptome data to identify the ion channels and transporters that were differentially expressed in jejunal and colonic SMCs. In jejunal SMCs, a total of 442 ion channel and transporter isoforms (258 ion channels, 169 ion transporters, and 15 coupled ion channels and transporters) were expressed (S8 Table). Likewise in colonic SMCs, a total of 447 ion channel and transporter isoforms (263 ion channels, 168 ion transporters, and 16 coupled ion channels and transporters) were expressed (S9 Table). Not surprisingly, calcium channels were the most highly expressed type of ion channel in both JSMCs and CSMCs (Fig 6A). Furthermore, hydrogen transporters were the most dominantly expressed type of transporter in JSMCs and CSMCs (Fig 6B). To identify the most abundantly expressed ion channel and transporter classes in SMCs as well as SMC-specific isoforms, a more detailed analysis was performed.

### Ion channels: calcium channels

**Fig 7:**
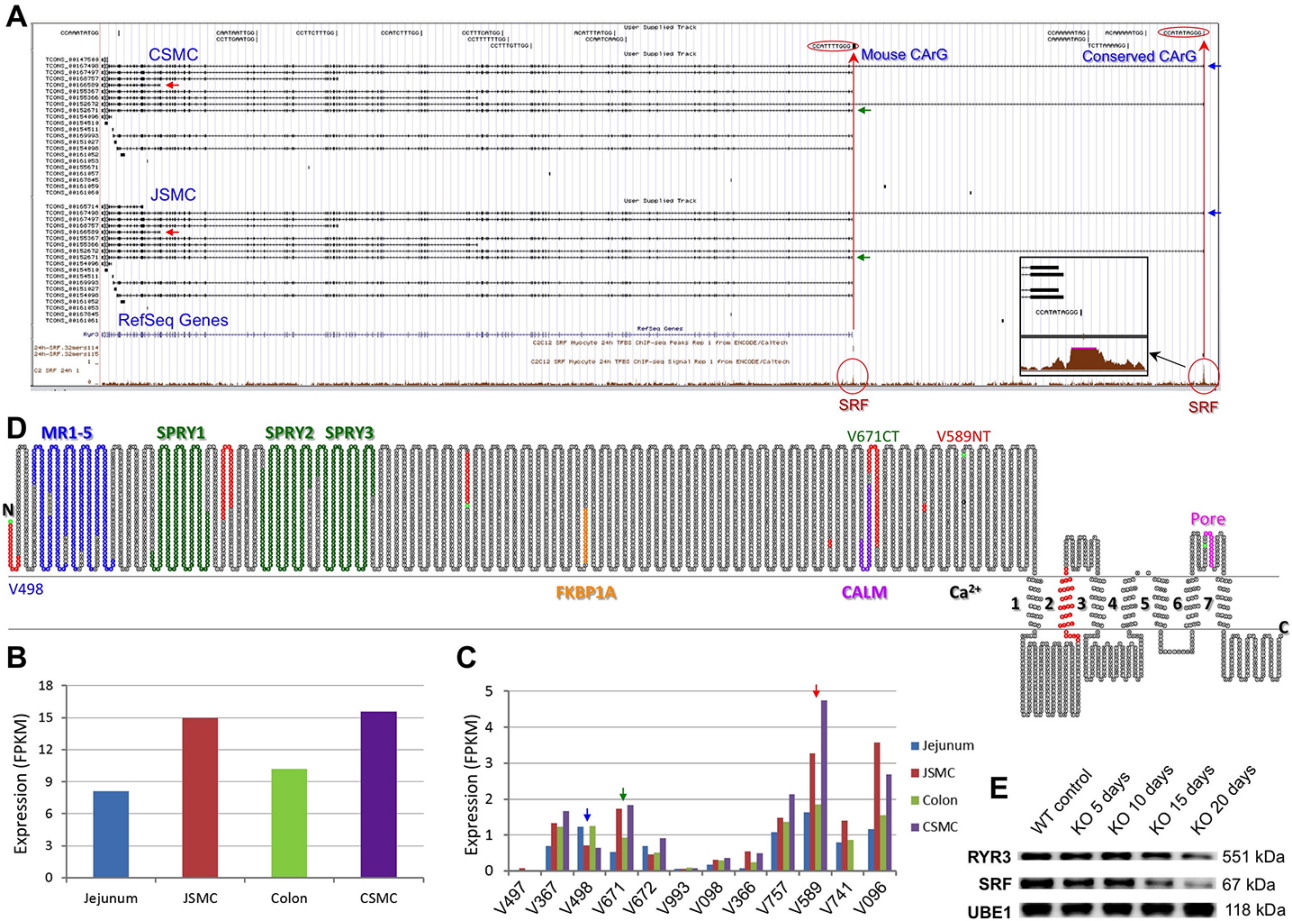
Identification of an SMC-specific Ryr3 regulated by SRF. (A) A genomic map view of *Ryr3* variants expressed in JSMCs and CSMCs. Three variants TCONS_00167498 (V498, blue), TCONS_00152671 (V671, green), and TCONS_00166589 (V589, red) are indicated by arrows. (B) Expression levels of total Ryr3 in SMCs. (C) Expression levels of each *Ryr3* vaiant in SMCs. Variants are arranged on the X-axis from longest (left) to shortest (right) in length. The three variants shown on A are also indicated by the color arrows. (D) A topological map of RYR3 variants. Each circle denotes the corresponding amino acid. Colors on amino acid sequence show distinct regions and domains: red, missing or inserted peptides from differentially spliced exons; green, start codons found in differentially spliced variants. Seven transmembrane domains 1–7 and a pore region (magenta) are shown. MR1-5 (blue), SPRY1-3 (dark green), FKBP1A (orange), CALM (purple), and a residue E (black) that is required for Ca^2+^ binding (Ca^2+^) are also indicated. The topology map was drawn based on the longest peptide variant V498. V671 is a C-terminal truncated variant (V671CT) that is missing Ca^2+^ binding and transmembrane domains after the CALM domain while V589 is an N-terminal truncated variant (V589NT) that is missing the N-terminal domains (N-ter to CALM domain). (E) Western blot showing that RYR3 protein expression decreased in jejunum SM of inducible SMC-specific *Srf* KO mice as SRF protein was depleted 5, 10, 15, and 20 days following tamoxifen administration. UBE1 was used as an endogenous control.

SMCs expressed several calcium channel isoforms that included voltage-dependent calcium channels [*Cacna1h* (T-type, Cav3.2); *Cacna1c* (L-type, Cav1.2); *Cacna1e* (R-type, Cav2.3); *Cacna1a* (P/Q-type, Cav2.1)] as well as calcium channel regulators, such as *Jph2* and *Ga6* (Fig 6C). Our transcriptome data showed that *Ryr3*, *Jph2*, *Cacna1e*, and *Cacna1c* were specifically expressed in JSMCs and CSMCs (Fig 6D). Furthermore, L-type (*Cacna1c* and *Cacna1d*) and T-type calcium channels (*Cacna1h* and *Cacna1g*) were also highly expressed in JSMCs and CSMCs (Fig 6E and 6F). Since the genes *Ryr3* and *Jph2* were the most specific to SMCs, we examined them in further detail (Fig 6D). *Ryr3* encodes a ryanodine receptor that functions to release calcium from intracellular stores and therefore, plays an essential role in triggering muscle contraction [29]. Analysis of the *Ryr3* gene with the genome browser revealed a SRF binding site on its promoter as well as on intron 1 (Fig 7A). Moreover, the SRF binding sites of the *Ryr3* gene contained two CArG boxes, one of which was located on the promoter and found to be conserved between mice and humans. Like other SRF-induced genes, *Ryr3* was predominantly expressed in JSMCs and CSMCs (Fig 7B), and there were approximately 12 *Ryr3* transcript variants, of which most were newly identified in this study (Fig 7A and 7C).

The transcriptional initiation sites for the long *Ryr3* transcripts TCONS_00167498 (16,179 bp) and TCONS_00152672 (15,870 bp) were located on the newly discovered exon 1, which was located ~170 kb upstream from exon 2 and was not present in the RefSeq gene sequence (NM_177652.2; Fig 7A). This *Ryr3* RefSeq gene sequence (NM_177652), which started at a cryptic alternative exon 1 near exon 2, was found to be in provisional status since it was missing the 5’ untranslated region. A *Ryr3* variant, which started on exon 1, was also found to be present in humans (NM_001036). The presence of a SRF binding site and CArG box in the proximal promoter region of exon 1 further validated its discovery and association with SRF (Fig 7A). The SRF binding site also contained a CArG box (CCATATAGGG) that was again conserved in mice and humans. Furthermore, an additional SRF binding site was located downstream at the alternative exon 1, which coincided with the transcriptional start sites of 5 *Ryr3* variants (TCONS_00167497, TCONS_00155367, TCONS_00152671, TCONS_00169993, and TCONS_00154098). The latter SRF binding site was also located in the proximal promoter region, which contained a CArG box (CCATTTTGGG) only found in mice.

As expected, the *Ryr3* variants were preferentially expressed in jejunal and colonic SMCs over their muscularis tissues (Fig 7B). Interestingly, however, there was significant variation in the expression levels of *Ryr3* variants between jejunal and colonic SMCs (Fig 7C). The most highly expressed *Ryr3* variant in both JSMCs and CSMCs was TCONS_00166589, which was a shorter variant (5,076 bp) containing only the 3’end of the full sequence.

To study the primary protein structures of *Ryr3* variants, the predicted peptide sequence of each RYR3 variant was obtained from their prospective open reading frame. The amino acid sequences of all RYR3 variants were aligned with each other to identify conserved domains and regions (S5 Fig). The full-length RYR3 peptide was TCONS_00167498 (V498, 4,858 aa), which consisted of MR1-5 domains, B30.2/SPRY1-3 domains, Repeat1-4 regions, FKBP1A, CALM, a Ca^2+^ binding residue, TM1-7 domains, and a pore region. The other eleven RYR3 variants were truncated either at the N-terminus or C-terminus. For example, the RYR3 variant TCONS_00152671 (V671CT) was truncated at the C-terminus and consisted of MR1-5 domains, B30.2/SPRY1-3 domains, Repeat1-4 regions, FKBP1A, and CALM but lacked the Ca^2+^ binding residue, TM1-7 domains, and the pore region. The most highly expressed RYR3 variant, TCONS_00166589 (V589NT), was truncated at the N-terminus and contained the Ca^2+^ binding residue, TM1-7 domains, and the pore region while missing the N-terminal domains and regions (Fig 7D).

In order to study the regulation of *Ryr3* gene by SRF, we examined *Ryr3* expression in the tamoxifen-inducible SMC-specific *Srf* KO mice. Successful KO of *Srf* expression was validated by Western blotting, which showed a progressive decline of SRF protein expression in jejunal SM apparent at 15 days following tamoxifen administration (Fig 7E). Likewise, RYR3 protein expression also decreased significantly after KO day 15, suggesting that SRF induces expression of RYR3 protein in the SMCs. UBE1 was used as the loading control since its expression levels did not change in the *Srf* KO SM. In contrast, GAPDH and ACTB could not be used as loading controls since their expression levels decreased in SM with *Srf* KO (S7 Table). Altogether, the data suggested that RYR3 was a newly discovered SRF-induced intracellular calcium ion release channel, which was predominantly expressed in JSMCs and CSMCs.

Junctophilin 2 (*Jph2*), the second most SMC-specific gene (Fig 6D), encodes a junctional membrane complex protein that regulates intracellular calcium release channels [30]. Not surprisingly, we found that *Jph2* also contained multiple SRF binding sites (Fig 4F), of which one was located on the promoter, one on intron 1, and two on intron 2 (S6A Fig). All four binding sites contained five CArG boxes, and four of them were conserved between mice and humans. Similar to *Ryr3*, *Jph2* was also found to be preferentially expressed in both JSMCs and CSMCs (S6B Fig). Moreover, we identified approximately six variants of the *Jph2* gene expressed in JSMCs and CSMCs, of which four were newly discovered in this study (S6A Fig). Two of the major variants included a long transcript (TCONS_00150012) that was 4,235 bp in length and a truncated transcript (TCONS_00152782) that was 1,663 bp in length (S6A and S6C Fig). The expression of JPH2 protein was also significantly decreased in *Srf* KO SM suggesting that its expression is induced by SRF (Fig 4F and S7 Table). This data suggested that JPH2 was also a newly discovered SRF-induced calcium channel modulator specifically expressed in SMCs.

### Ion channels: potassium, cation, chloride, and sodium channels

Interestingly, we also found that potassium, cation, chloride, and sodium channel isoforms were expressed in SMCs at levels that were comparable to each other but approximately 50% lower than that of calcium channels (Fig 6A). Most potassium channel isoforms were expressed at low levels in SMCs. However, there were several other potassium channel genes that were highly and specifically expressed in SMCs: *Kcnj8* (Kir6.1), *Abcc9* (Sur2), and *Kcnmb1* (BKβ1; S7A Fig). Although *Kcnip2* (Kv channel interacting protein 2) was also highly expressed in JSMCs, *Kcnf1* (Kv5.1) expression was most specific to SMCs (S7B Fig). Among cation channels, *Aoc3* (VAP1) was the most abundantly expressed in SMCs (S7C Fig). Although there were several SMC-specific cation channels, *Trpv2* and *Trpc2* were the most highly expressed in SMCs (S7D Fig).

In regards to chloride channels, the chloride channel regulator *Fxyd1* was the most abundantly expressed in SMCs (S7E Fig), but *Gabrb2* (ligand-gated chloride channel) was the most SMC-specific (S7F Fig). As expected, sodium channel isoforms were expressed at much lower levels than other cation channels in SMCs (Fig 6A). Interestingly, *Scn1b* (voltage-gated sodium channel type 1β) and *Scnn1a* (nonvoltage-gated sodium channel type 1α) were the most highly expressed sodium channel in both JSMCs and CSMCs (S7G Fig). However, *Scn2a1* (voltage-gated sodium channel type 2α) was most SMC-specific (S7H Fig).

### Transporters: hydrogen and sodium transporters

Strikingly, we found that the hydrogen transporter isoforms were the main class of transporters expressed in SMCs (Fig 6B), and the most highly expressed isoforms were preferentially expressed in JSMCs than in CSMCs (S8A Fig). Among the hydrogen transporters, *Atp5b* (proton-transporting ATPase) was the most highly expressed and SMC-specific isoform (S8A and S8B Fig).

In contrast to hydrogen transporter isoforms, sodium transporter isoforms were expressed at much lower levels (Fig 6B). Since sodium transporters are more characteristic of intestinal epithelial cells [31], this finding was anticipated since SMCs of the SM were separated from the mucosa during the purification process via FACS. Most of these sodium transporters were found to be co-transporters of other ions including potassium (S8 and S9 Tables), and the most highly expressed hydrogen transporters in SMCs were *Atp1a1* (sodium/potassium exchanging ATPase) and *Slc24a3* (NCKX3, sodium/potassium/calcium exchanger; S8C Fig). Furthermore, *Slc6a17* (sodium-dependent neurotransmitter transporter) was the most SMC-specific gene (S8D Fig).

## Discussion

In this study, we identified the collective transcriptome and CArGome of SMCs pooled from the small and large intestines of approximately 40 mice. In doing so, we uploaded and implemented our data into a publicly accessible browser via the UCSC genome database. The transcriptomes included all genetic isoforms and transcriptional variants expressed in the pooled mouse jejunal and colonic SMCs.

The genome browser is an interactive database that can be used to search for any expressed gene transcript in SMCs. Furthermore, it can be used to analyze the expression and regulation of individual genes by integrating with the abundant bioinformatics data in the UCSC genome database [13]. In addition, the browser makes it possible to use all of the publically available genome bioinformatics data, e.g., ENCODE, which is within the database.

RNA-seq is a powerful sequencing technology capable of generating a genome scale transcriptome quantitatively with single base resolution [32]. However, in order to identify the entire transcriptome, including mRNAs expressed in low levels, acquisition and sequencing of at least 100 million reads is required. Since we could isolate only a limited numbers of primary GI SMCs per mouse by FACS, we pooled together SMCs isolated from 40 male and female mice for mRNA isolation (N = 40). We obtained over 150 million reads from the pooled mRNA sample (N = 1). Therefore, the expression profile in the transcriptome data represents the average expression level of 40 mice for each gene. The caveat to this methodology is that the expression profile of isoforms expressed in low levels may not represent SMC mRNAs that were consistently expressed in SMCs of all 40 mice. Therefore, the individual isoforms require experimental validation in SMCs.

We identified approximately 16,000 genes in the transcriptome, which represented 64% of the approximately 25,000 total genes encoded within the mouse genome. In addition, each gene expressed multiple alternative transcriptional isoforms (an average of three variants per gene) culminating in approximately 46,000–55,000 genetic variations. All variants were generated from alternative start sites and exons, of which most appeared to be specific to SMCs. In humans, about 95% of multi-exonic genes are known to be alternatively spliced [33], and the alternative exons provide biodiversity of proteins [34].

A transcriptome is a blueprint for gene expression and function, and identification of all isoforms and transcriptional variants of genes expressed in SMCs is vital to understanding their molecular functions. In this regard, our discovery that SMCs express multiple isoforms of gene families, of which one may be dominant, should be considered for gene expression studies. Our SMC genome browser and data will assist in the identification of candidate isoforms (subunits) of protein complexes, such as ion channels, relevant to SMCs. Furthermore, we have shown that each gene is differentially transcribed into multiple variants (S2 and S3 Tables). These transcriptional variants had not been previously considered in functional studies, which mostly utilized only the reference genes available in genomic databases. Importantly, these alternative transcripts of variants can lead to amino acid sequence changes via deletions and insertions of alternative exons (e.g., Ryr3 in Fig 7). The different expression patterns of variants in SMCs may be responsible for their distinctive cellular and molecular functions. This transcriptome data, which is available through the interactive SMC transcriptome browser, contains all transcriptional variants, expression levels, and exon maps of genes expressed in SMCs. As such, it will assist researchers in identifying alternatively and dominantly expressed transcripts for genes of interest in future functional and molecular studies of SMCs.

In addition, the SMC transcriptome browser can also facilitate analysis of protein-coding and non-coding genes in relation to SRF binding sites and CArG boxes mapped to the genome. Therefore, further identification and analysis of as yet unrecognized CArG-containing genes in the context of diseases that involve SRF and the SM could provide greater insights into pathophysiological mechanisms. The growing number of long non-coding RNAs that are being discovered is also of great interest because of their role in regulation of gene transcription, and its incorporation into our transcriptome data as a parallel analysis could provide even greater insight into the regulatory mechanisms of gene expression in SMCs. Given the diversity of cells that express SRF, it will be critical to perform ChIP-seq studies in other cell types and in stress-induced conditions to further validate the computationally predicted CArGome. Finally, our study highlights the need for a fully defined human CArGome that can be superimposed onto this dataset along with CArG-SNPs, some of which have already been reported [17].

The SRF controls different programs of gene expression across several distinct cell types, and its absence has profound phenotypic consequences [35]. Proliferating myoblasts (C2C12), whether cardiac or skeletal muscle, share a similar program of SRF-dependent gene transcription with SM [20]. These genes include*Acta2*, *Tagln1*, *Cnn1*, *Lmod1*, *Csrp1*, *Fhl2*, and *Kcnmb1*. Moreover, SRF is a master regulator of the actin cytoskeleton [20], in which hundreds of genes common to many cell types, including SMC, are driven by CArG-SRF. The ChIP-seq data utilized in this study may have limitations since it was acquired from skeletal muscle cells rather than mature SMCs. However, since these cells share a common pathway of muscle gene programing by CArG-SRF, the SRF binding site data from C2C12 myoblasts can be cautiously extrapolated to the SMC lineage.

By integrating with the ENCODE database, this study also demonstrated that histone modifications (H3k4m3, H3k27a and H3k27m3) on gene transcripts are an excellent means of identifying SMC-specific markers (Fig 5). These cell-specific markers, which have been identified through our analyses, provide new tools for studying the mature SMC phenotype.

SMCs display phenotypic plasticity and therefore, can dedifferentiate into a myofibroblast-like synthetic phenotype in pathological conditions that stimulate hyperplasia as well as in cell culture conditions [2]. During their transition to a proliferative state, SMCs lose expression of contractile proteins. Several putative SMC markers have been used in the evaluation of the SMC phenotype. However, it is still unclear which of these markers are expressed exclusively in differentiated primary SMCs because most studies of SMC gene expression and signaling pathways have been based on SMCs grown in culture (60,910 papers published in PubMed Central, as of 4/29/2015), for which the standard of identification has been *Acta2*. Moreover, tissue engineering efforts for GI muscle have also relied on this marker [36], which has not been thoroughly validated *in vivo*. Therefore, very little is still known about the actual phenotype of the cells that are being grown for tissue engineering and signal transduction studies.

The most specific markers for primary SMCs isolated from mouse intestinal SM were *Actg2*, *Myh11*, and *Acta2* (Fig 5A). Both *Actg2* and *Acta2* are smooth muscle specific isoforms of actin whose expression levels in JSMCs and CSMCs were much higher than that of *Myh11* (Fig 5B and 5C). The discrepancy between the expression levels of these genes may be linked to juxtapositioning with SRF binding sites. For instance, multiple CArG boxes conserved between mice and humans are located in promoter regions of *Actg2* (4 CArG boxes), *Myh11* (3 CArG boxes), and *Acta2* (4 CArG boxes). Interestingly, CArG boxes in promoters of *Actg2* [-313 bp, -254 bp, -82 bp, and -45 bp from the transcription start site (TSS)] and *Acta2* [two TSS: 1^st^ TSS (-121 bp, -71 bp) and 2^nd^ TSS (-290 bp, -117 bp)] are located much closer to TSS than those of *Mhy11* (-1,117 bp, -1,041 bp, and -922 bp). This observation suggests that the number of CArG boxes and their proximity to TSS or RNA polymerase II binding sites may influence the expression levels of CArG-dependent genes. Furthermore, analysis of the histone markers revealed that *Cnn1*, *Mylk*, *Tpm2*, *Tpm1*, *Des*, and *Myh11* are the most distinctive differentiation markers (Fig 5D). These marker genes offer a newly refined set of tools to improve the phenotypic characterization and mechanical performance of engineered SMCs in culture. Furthermore, the newly discovered markers may assist with more precise characterization of SMCs in pathological conditions *in vivo*.

Our transcriptome analysis also revealed a variety of ion channels and transporters that are abundantly expressed in SMCs thus, providing a unique overview of the physiologic composition of SMCs. A large number of ion channels and transporters (442 in JSMCs and 447 in CSMCs) were expressed in SMCs. However, there were many more expressed ion channels (253–263) than ion transporters (169–168; S8 and S9 Tables) indicating that SMC excitability is regulated by a complex coordinated effort of numerous ion channels. Within the ion channel family, calcium channels were the most abundantly expressed in SMCs, and the predominance of calcium channel expression is consistent with the current paradigm for excitation-contraction coupling, which is primarily regulated by Ca^2+^ via calcium channels [7]. Furthermore, our finding that the L-type calcium channel (*Cacna1c*) exhibited the highest levels of expression of all ion channels in SMCs implicates its pivotal role in SM contraction. In a separate set of experiments, we also found that expression of *Cacna1c* was indirectly regulated by SRF via myotonic dystrophy protein kinase (DMPK; manuscript in submission). However, due to the influence of post-transcriptional processing mechanisms, caution should be exercised in extrapolating the mRNA expression data to actual differences in ion channel function.

The two genes, whose expression was most specific to SMCs, were the ryanodine receptor (*Ryr3*) and junctophilin (*Jph2*; intracellular calcium release channel regulator). The SMC specificity of their expression appeared to occur through SRF-binding of CArG boxes. SMCs expressed three isoforms of the ryanodine receptor (*Ryr1*-*3*), of which *Ryr2* and *Ryr3* were most highly expressed (S4 and S5 Tables). Using the SMC genome browser, we discovered that *Ryr3* is also a target of SRF and associated with a CArG box. SMCs were also found to express four junctophilin isoforms (*Jph1*-*4*), of which *Jph2* was the most highly expressed in the SMCs.

*Ryr2* encodes a Ca^2+^ induced Ca^2+^ release channel, which is localized to the junctional membrane complex between the plasma membrane and endoplasmic/sarcoplasmic reticulum [37], and the JPH2 protein is an essential component of the junctional complex [38]. The latter protein is known to regulate the activities of ryanodine receptors [39], L-type calcium channels [40], and TRPC channels [41] in cardiac myocytes and skeletal muscle, but there are no known reports of this gene in SM. In this study, we found that *Jph2* is the predominant isoform expressed in primary SMCs. Further functional studies of the interactions between JPH2 and the aforementioned ion channels will be required in order to confirm its role in the regulation of excitation-contraction coupling in SMCs.

The modulatory effects of SRF on its targeted genes that were identified in this study were validated with the proteomics data obtained from the *Srf* KO mice (S7 Table). This *in vivo* validation confirmed that our interactive SMC genome and CArGome browser may serve as a powerful tool for identifying SRF-regulated genes and that it can be applied to other regulators of SMC gene expression that are readily available in the database.

## Supporting Information

S1 Extended Materials and Methods (DOCX)

S1 Fig Confirmation of cell markers expressed in SMCs. (A) *Myh11* (SMCs), (B) *Kit* (ICC), (C) *Pdgfra* (PDGFRα^+^ cells), and (D) *Uchl1* (PGP9.5, neuronal cells).
(TIF)

S2 Fig Identification and validation of transcriptional variants of *Srf* expressed in colonic and jejunal SMCs. (A) A genomic map of *Srf* variants showing three mRNA transcripts with alternative start sites, an antisense RNA (asRNA), and a noncoding RNA (ncRNA) that aligns upstream from the promoter. Exons are numbered as E1-7, and the three variable forms of exon1 are indicated as E1a-c. SRF binding sites, conserved CArG boxes, a CpG island, and H3K4me3 (RNA polymerase II binding site) are shown. One SRF binding site is located on the promoter, and another is located on intron 2. Each SRF binding site contains one to three CArG boxes that are conserved between humans and mice. (B) Expression levels (FPKM) of total *Srf* mRNA in colonic and jejunal SMCs. (C) Expression levels (FPKM) of each Srf variant in colonic and jejunal SMCs. (D) PCR validation of *Srf* variants with different initiation sites in SMCs and SMs of jejunum and colon. NTC is non-template control. Primer sets were designed from variant exons in E1a-c (see S11 Table for primer sequences).
(TIF)

S3 Fig Identification and validation of transcriptional variants of *Cnn1* expressed in colonic and jejunal SMCs. (A) A genomic map of *Cnn1* variants showing five mRNA transcripts with alternative start sites. Exons are numbered E1-7. E1a-d, E2 (L/S), and E7 are variable. (B) Expression levels (FPKM) of total *Cnn1* mRNA in colonic and jejunal SMCs. (C) Expression levels (FPKM) of individual *Cnn1* variants in colonic and jejunal SMCs. (D) PCR validation of Cnn1 variants with different initiation sites in SMCs and SMs of jejunum and colon. NTC is non template control. Primer sets were designed from variant exons in the regions in E1 and E2 (see S11 Table for primer sequences).
(TIF)

S4 Fig Identification of CArG boxes in SRF binding sites of genes *Tpm1*, and *Tpm2*, *Cnn1*. (A-C) A genomic map of *Tpm1*, *Tpm2*, and *Cnn1* mRNA variants expressed in jejunal SMC showing conserved CArG boxes found within SRF binding sites from publically available data (C2C12). Binding sites of RNA Polymerase (Pol) II is shown in parallel with the variants and SRF binding sites.
(TIF)

S5 Fig Alignment of predicted amino acid sequences of RYR3 transcriptional variants. The open reading frame was identified for each transcriptional variant, and predicted amino acid sequences were aligned together. Seven transmembrane domains (TM1-7), a pore region, MR1-5, B30.2/SPRY1-3, FKBP1A, Repeat 1–4, CALM, and a residue E that is required for Ca^2+^ binding (Ca^2+^) are annotated along the amino acid sequences. Colors on the amino acid sequences designate start codons (green) and missing or inserted peptides (red) from differentially spliced or alternatively initiated exons.
(DOCX)

S6 Fig Identification of an SMC-specific Jph2 regulated by SRF. (A) A genomic map view of *Jph2* variants expressed in JSMCs and CSMCs. Six *Jph2* transcriptional variants are expressed in SMCs. There are four SRF binding sites on the promoter region, intron 1, and intron 2. Each SRF binding site contains one to two CArG boxes that are either mouse-specific or conserved between humans and mice. (B) Total expression levels (FPKM) of *Jph2* in SMCs. (C) Expression levels (FPKM) of individual *Jph2* variants in SMCs.
(TIF)

S7 Fig Identification of potassium, cation, chloride, and sodium channel isoforms expressed in SMCs. (A) Potassium channel isoforms enriched in JSMCs and CSMCs. (B) SMC-specific potassium channel isoforms. (C) Cation channel isoforms enriched in JSMCs and CSMCs. (D) SMC-specific cation channel isoforms. (E) Chloride channel isoforms enriched in JSMCs and CSMCs. (F) SMC-specific chloride channel isoforms. (G) Sodium channel isoforms enriched in JSMCs and CSMCs. (H) SMC-specific sodium channel isoforms. Cell specificity was determined by comparative analysis of gene expression profiles among SMCs, ICC, and PDGFRα^+^ cells.
(TIF)

S8 Fig Identification of hydrogen and sodium transporter isoforms expressed in SMCs. (A) Hydrogen transporter isoforms enriched in JSMCs and CSMCs. (B) SMC-specific hydrogen transporter isoform. (C) Sodium transporter isoforms enriched in JSMCs and CSMCs. (D) SMC-specific sodium transporter isoforms. Cell specificity was determined by comparative analysis of gene expression profiles among SMCs, ICC, and PDGFRα^+^ cells.
(TIF)

S1 Table Summary of SMC transcriptomes obtained from RNA-seq. (XLSX)

S2 Table Expression levels of transcriptional variants in jejunal SMCs. (XLSX)

S3 Table Expression levels of transcriptional variants in colonic SMCs. (XLSX)

S4 Table Expression levels of genes in jejunal SMCs. (XLSX)

S5 Table Expression levels of genes in colonic SMCs. (XLSX)

S6 Table A list of SRF binding sites associated with CArG boxes, CpG islands, and jejunal SMC transcripts. (XLSX)

S7 Table A list of SRF-regulated proteins identified in the jejunal smooth muscle from SMC-specific *Srf* knock mice. (XLSX)

S8 Table Expression levels of ion channel and transporter genes in jejunal SMCs. (XLSX)

S9 Table Expression levels of ion channel and transporter genes in colonic SMCs. (XLSX)

S10 Table Sequence permutations of CArG Boxes in the CArGome. (DOC)

S11 Table Oligonucleotides used in this study. (XLS)
