# Supplementary material for: Smooth Muscle Cell Genome Browser: Enabling the Identification of Novel Serum Response Factor Target Genes
Source: PLoS One. 2015 Aug 4;10(8):e0133751. doi: 10.1371/journal.pone.0133751 (PMC4524680; doi:10.1371/journal.pone.0133751)
Supplement: S1 Extended Materials and Methods — (DOCX) [file pone.0133751.s001.docx]

# S1 Extended Materials and Methods

**Construction of RNA-seq libraries and next-gen sequencing**

Four RNA-Seq libraries (jejunal smooth muscle tissue, jejunal SMCs, colonic smooth muscle tissue, colonic SMCs) were generated using Illumina’s sample preparation Kit (Illumina) according to manufacturer’s instructions. mRNAs were extracted using Sera-mag Magnetic Oligo(dT) Beads (Thermo Fisher). The purified mRNA samples were fragmented with a divalent cation solution. The fragmented mRNA samples were then subjected to first strand cDNA synthesis using reverse transcription with random primers, followed by second strand cDNA synthesis using DNA Polymerase I and RNase H. The double-stranded cDNA fragments were then end-repaired with T4 DNA Polymerase and Klenow DNA Polymerase. An “A” base was added to the blunt end of the cDNA fragments prior to adaptor ligation using the Klenow fragment. The adenylated cDNAs were purified with a MinElute PCR Purification Kit (QIAGEN) before used for adaptor ligation. Illumina’s paired-end oligo adapters were then added to the cDNA fragments with T4 ligase, followed by purification using a QIAquick PCR Purification Kit (QIAGEN). The library was eluted in 10 µl of Nulease-free water followed by purification on a 2% agarose gel. A gel with 250±25 bps was excised and the cDNAs were eluted using a QIAquick Gel Extraction Kit (QIAGEN). The eluted cDNAs were then enriched by 18 cycles of PCR, followed by gel purification. The recovered cDNAs were precipitated and quantified on Nanodrop and on TBS-380 mini-fluorometer (Turner Biosystems) using Picogreen dsDNA quantization reagent (Invitrogen). The concentration of the sample was adjusted to ~10 nM. The purified cDNA libraries were used for cluster generation on Illumina’s Cluster Station and then sequenced on Illumina HiSeqTM 2500 following vendor’s instruction at LC Sciences (2 samples/lane with Paired-end sequencing).

**Bioinformatics data analysis**

Raw sequencing intensities were extracted and the bases were identified using Illumina’s Real-time Analysis (RTA) software, followed by sequence quality filtering. The extracted sequencing reads were saved as a pair of fastq files for the first and second read. Filtered sequencing reads were aligned against the reference genome (UCSC mm9) using TopHat v1.4.1 software. Reads that did not directly map to the genome were used to identify potential exon-exon junctions. The result of sequence alignment was saved in the BAM format and further processed using Cufflinks 2.0.2 for transcript assembly. The expression level was estimated and presented in fragments per kilobase of transcript per million fragments mapped (FPKM). The transcripts, along with their associated exons, were documented in the GTF format. The assembled transcript GTF file was then annotated by comparison to the known reference using the Cuffcompare module of Cufflinks. For multiple groups of samples, the Cuffdiff module of Cufflinks was used for differential expression analysis. The FPKM value for each gene was used to compare the transcriptome and to identify the genes enriched in each population of the SMCs.

Gene ontology terms for each gene were retrieved from the UCSC genome database [[1](#_ENREF_1)] and used to analyze cell-specific genes including ion channels and transporters. Expression levels of splice variants for cell marker genes were analyzed and a transcriptome map view for each gene was generated on the genome browser. An open reading frame for each variant was predicted using ORF Finder at National Center for Biotechnology Information (NCBI) [[2](#_ENREF_2)] and the deduced amino acid sequence was aligned using ClustalW2. [[3](#_ENREF_3)] Domains and functional regions for protein were obtained in UniProtKB. [[4](#_ENREF_4)] Protein topology was generated using TOPO2 (http://www.sacs.ucsf.edu/cgi-bin/open-topo2.py/).

**Mouse CArGome**

A custom Perl script was created to identify CArG elements genome-wide (CArGome) in *mus* *muscularis*. The repeat-masked mm9 genome assembly for *mus* *muscularis* consisting of chromosomes 1 to 19, X, and Y was obtained from the UCSC genome database [[5](#_ENREF_5)]. Each aligned chromosome sequence was interrogated using the Perl script to identify CArG elements using regular expressions that match the pattern CCW_6_GG (consensus CArG box), and allowed for 1 bp deviation (CArG-like box) as previously reviewed. [[6](#_ENREF_6)] The specific regular expressions matched are listed in S10 Table. Conserved CArG boxes were identified by comparing between mouse CArGome and human CArGome. [[7](#_ENREF_7)] Mouse and conserved CArG boxes were documented in the BEB format.

**Data access**

SMC transcriptome and CArGome data were deposited in the custom track of the UCSC genome database [[1](#_ENREF_1)]. Complied SMC transcriptome files (GTF) and CArG boxes (BEB) are available at public links: SM_Jejunum.gtf, <http://dx.doi.org/10.6084/m9.figshare.1436176>; SM_Colon.gtf, <http://dx.doi.org/10.6084/m9.figshare.1436177>; SMC_Jejunum.gtf, <http://dx.doi.org/10.6084/m9.figshare.1436178>; SMC_Colon.gtf, <http://dx.doi.org/10.6084/m9.figshare.1436179>; CArG_Mouse.bed, <http://dx.doi.org/10.6084/m9.figshare.1437667>; CArG_Conserved.bed, <http://dx.doi.org/10.6084/m9.figshare.1437662>;. To build the genome browser, 1) go to [Add Custom Tracks on UCSC Genome Browser](http://genome.ucsc.edu/cgi-bin/hgCustom?hgHubConnect.destUrl=..%2Fcgi-bin%2FhgTracks&clade=mammal&org=Mouse&db=mm9&position=chr1%3A4%2C797%2C973-4%2C836%2C816&hgt.positionInput=enter+position%2C+gene+symbol+or+search+terms&hgt.suggestTrack=knownGene&hgsid=368240703_p92AXqmz0Aa9Kz1cAqQ6KP97Aw0C), 2) upload each file using Browse and Submit, 3) click User Track and change ‘User Track’ to a file name, and 4) after uploading all the files, click go to genome browser. The browser contains transcriptome menus (SM Jejunum, SM Colon, SMC Jejunum, and SMC Colon) and CArG box menus (CArG Mouse and CArG Conserved) on the “Custom Tracks”. Each menu has different display options (hide, dense, squish, pack, or full).The custom built browser was named “UCSC Smooth Muscle Genome Browser”, which is available at <http://medicine.nevada.edu/physio/transcriptome> (it requires Google Chrome and takes ~1 minutes to upload the large files).

The abbreviated instructions are as follows: 1) To search transcriptional variants of a gene, type in the gene symbol, and click “go.” 2) Under “Custom Tracks,” select the view option (e.g., “full”) for type of sample (e.g., “SMC_Jejunum”), and click “refresh.” 3) Select the bioinformatics data of interest (e.g., click on “full” under “RefSeq Genes” in “Genes and Gene Predictions” and/or “Caltech TFBS” under “Expression and Regulation” for SRF binding sites), and then click “refresh.” 4) Select options in each bioinformatics data (e.g., click “Caltech TFBS,” select “SRF,” change the display mode to “full,” and click “Submit”). 5) Click “configure” to optimize views (change image width and text size).

The RNA-seq data from this study have been also submitted to the NCBI: GSM1388412, SM Jejunum; GSM1388413, SM Colon; GSM1388406, SMC Jejunum; GSM1388407, SMC Colon.

**References**

1. Meyer LR, Zweig AS, Hinrichs AS, Karolchik D, Kuhn RM, Wong M, et al. (2013) The UCSC Genome Browser database: extensions and updates 2013. Nucleic Acids Res 41: D64-69.

2. Sayers EW, Barrett T, Benson DA, Bolton E, Bryant SH, Canese K, et al. (2012) Database resources of the National Center for Biotechnology Information. Nucleic Acids Res 40: D13-25.

3. McWilliam H, Li W, Uludag M, Squizzato S, Park YM, [Buso N](http://www.ncbi.nlm.nih.gov/pubmed/?term=Buso%20N%5BAuthor%5D&cauthor=true&cauthor_uid=23671338), et al. (2013) Analysis Tool Web Services from the EMBL-EBI. Nucleic Acids Res 41: W597-600.

4. Boutet E, Lieberherr D, Tognolli M, Schneider M, Bairoch A (2007) UniProtKB/Swiss-Prot. Methods Mol Biol 406: 89-112.

5. Waterston RH, Lindblad-Toh K, Birney E, Rogers J, Abril JF, Agarwal P, et al. (2002) Initial sequencing and comparative analysis of the mouse genome. Nature 420: 520-562.

6. Miano JM, Long X, Fujiwara K (2007) Serum response factor: master regulator of the actin cytoskeleton and contractile apparatus. Am J Physiol Cell Physiol 292: C70-81.

7. Benson CC, Zhou Q, Long X, Miano JM (2011) Identifying functional single nucleotide polymorphisms in the human CArGome. Physiol Genomics 43: 1038-1048.
