# Supplementary figures and images for: Smooth Muscle Cell Genome Browser: Enabling the Identification of Novel Serum Response Factor Target Genes
Source: PLoS One. 2015 Aug 4;10(8):e0133751. doi: 10.1371/journal.pone.0133751 (PMC4524680; doi:10.1371/journal.pone.0133751)

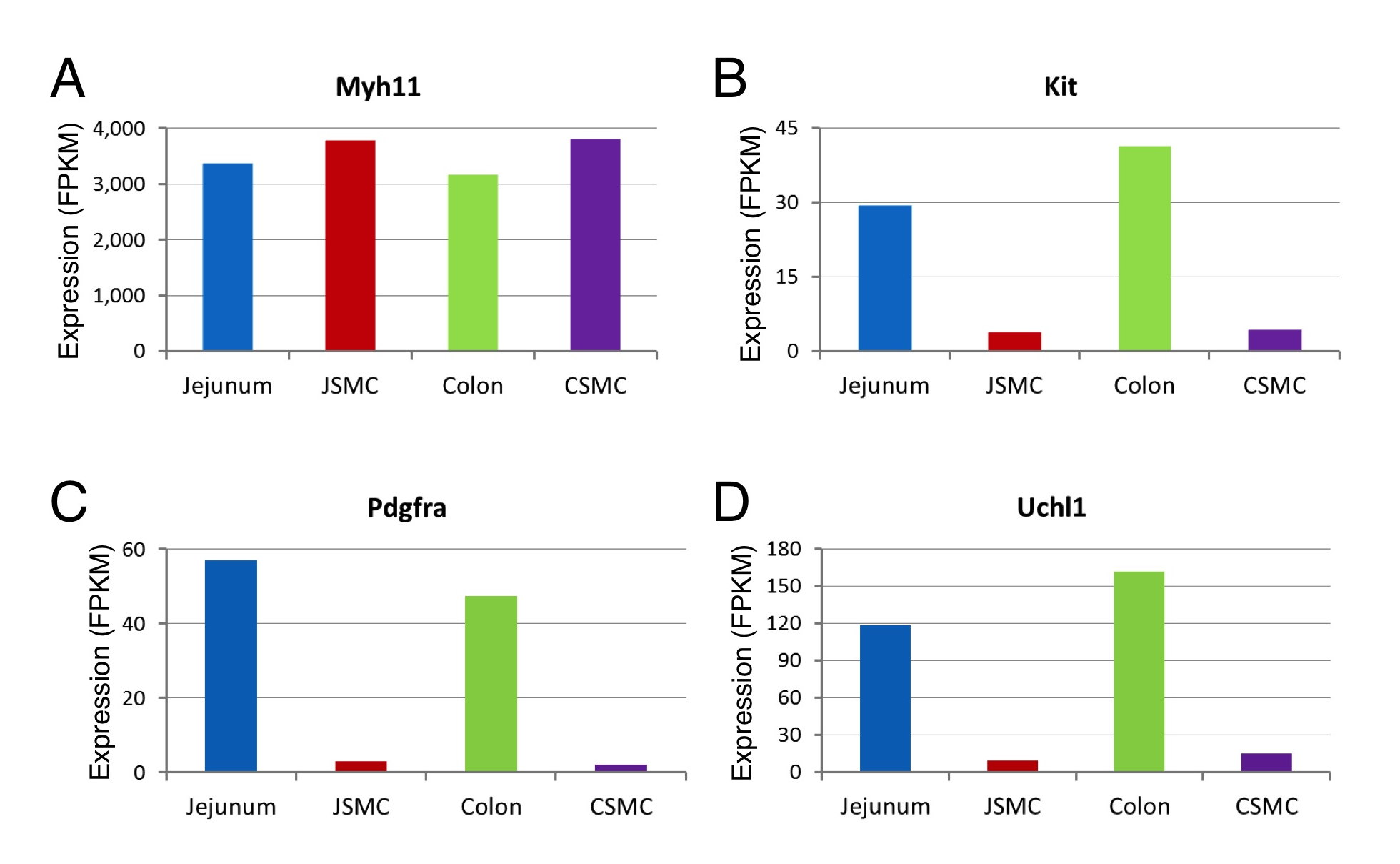

Supplement: S1 Fig — (A) Myh11 (SMCs), (B) Kit (ICC), (C) Pdgfra (PDGFRα+ cells), and (D) Uchl1 (PGP9.5, neuronal cells). (TIF) [file pone.0133751.s002.tif]

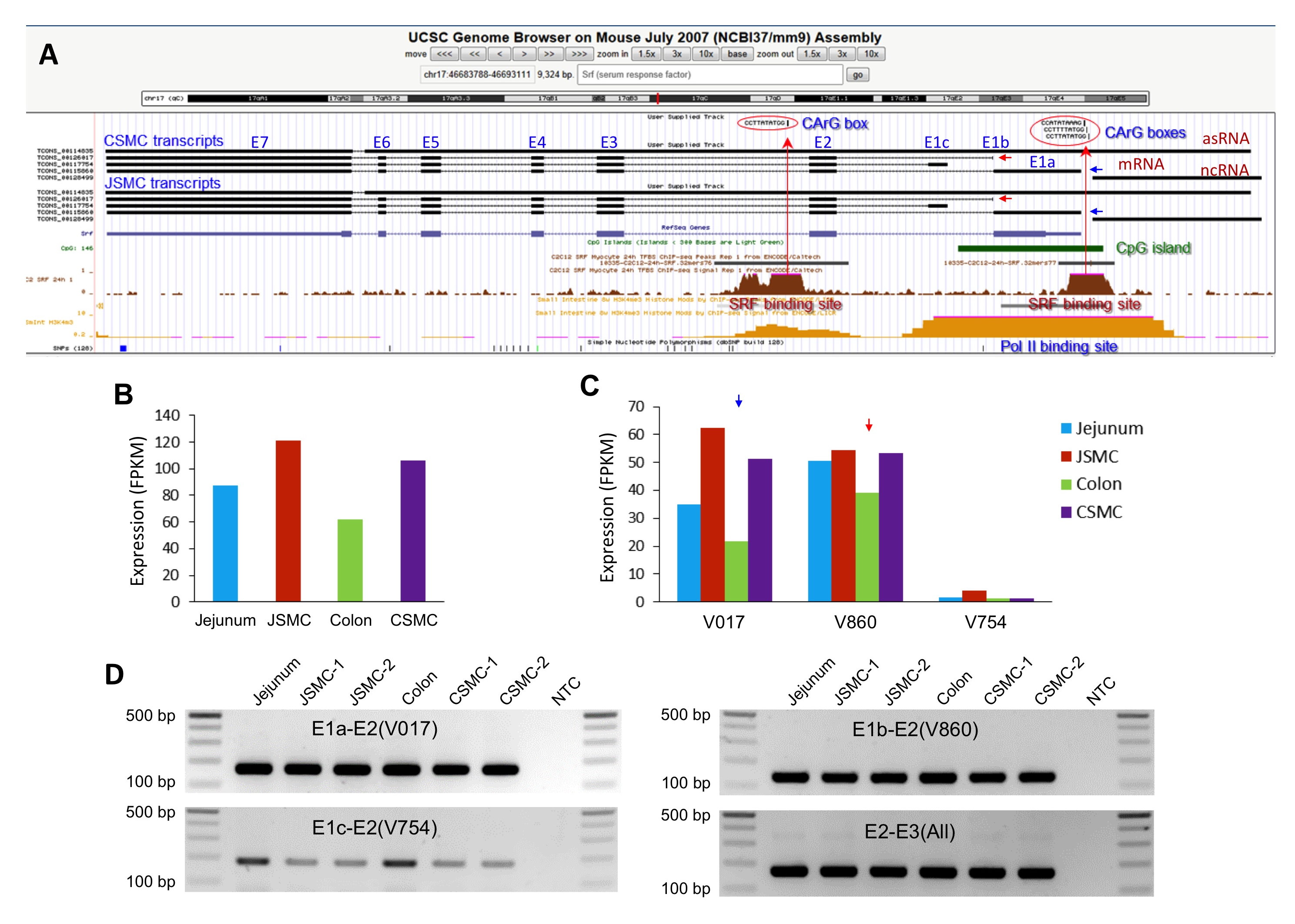

Supplement: S2 Fig — (A) A genomic map of Srf variants showing three mRNA transcripts with alternative start sites, an antisense RNA (asRNA), and a noncoding RNA (ncRNA) that aligns upstream from the promoter. Exons are numbered as E1-7, and the three variable forms of exon1 are indicated as E1a-c. SRF binding sites, conserved CArG boxes, a CpG island, and H3K4me3 (RNA polymerase II binding site) are shown. One SRF binding site is located on the promoter, and another is located on intron 2. Each SRF binding site contains one to three CArG boxes that are conserved between humans and mice. (B) Expression levels (FPKM) of total Srf mRNA in colonic and jejunal SMCs. (C) Expression levels (FPKM) of each Srf variant in colonic and jejunal SMCs. (D) PCR validation of Srf variants with different initiation sites in SMCs and SMs of jejunum and colon. NTC is non-template control. Primer sets were designed from variant exons in E1a-c (see S11 Table for primer sequences). (TIF) [file pone.0133751.s003.tif]

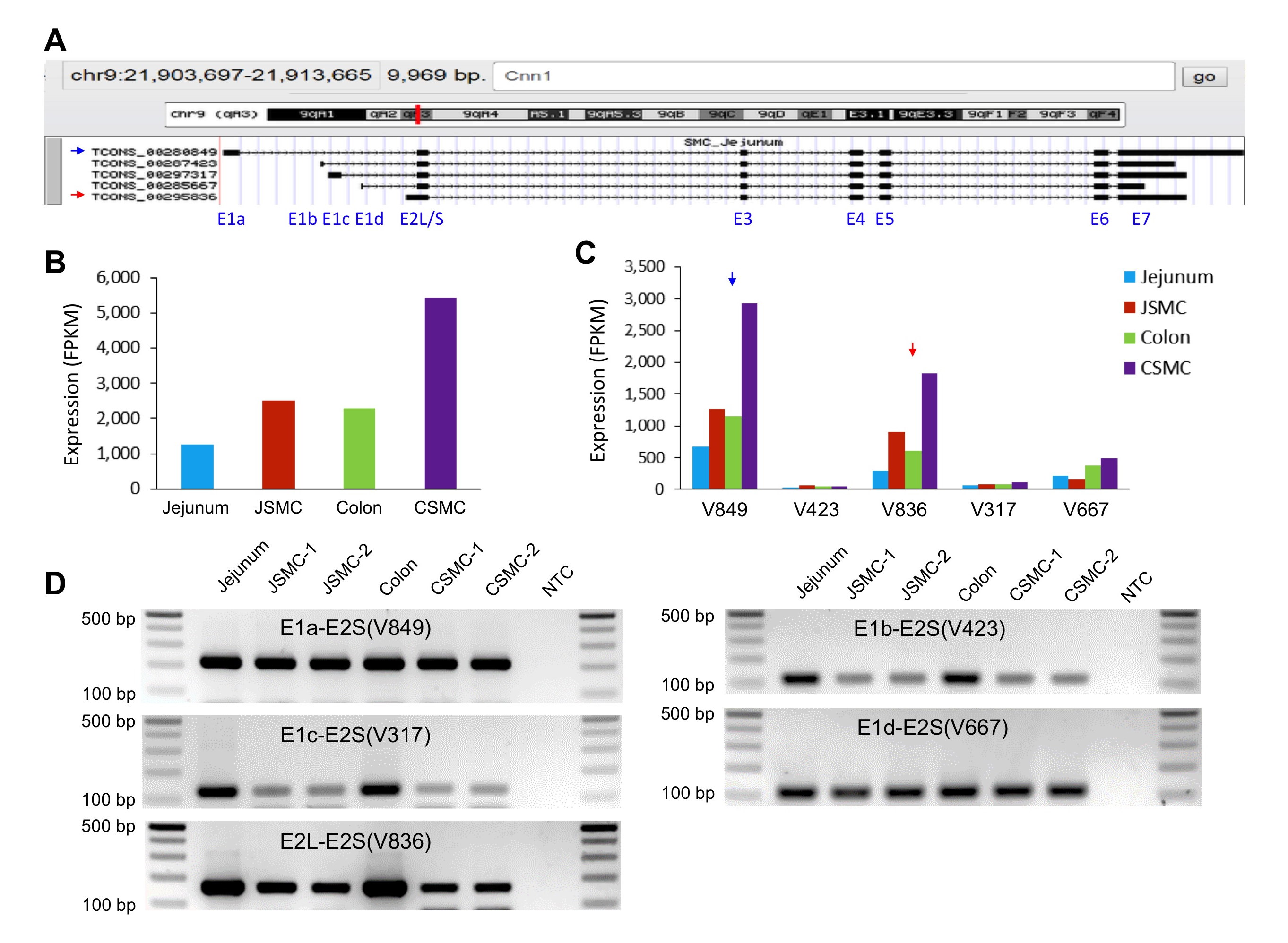

Supplement: S3 Fig — (A) A genomic map of Cnn1 variants showing five mRNA transcripts with alternative start sites. Exons are numbered E1-7. E1a-d, E2 (L/S), and E7 are variable. (B) Expression levels (FPKM) of total Cnn1 mRNA in colonic and jejunal SMCs. (C) Expression levels (FPKM) of individual Cnn1 variants in colonic and jejunal SMCs. (D) PCR validation of Cnn1 variants with different initiation sites in SMCs and SMs of jejunum and colon. NTC is non template control. Primer sets were designed from variant exons in the regions in E1 and E2 (see S11 Table for primer sequences). (TIF) [file pone.0133751.s004.tif]

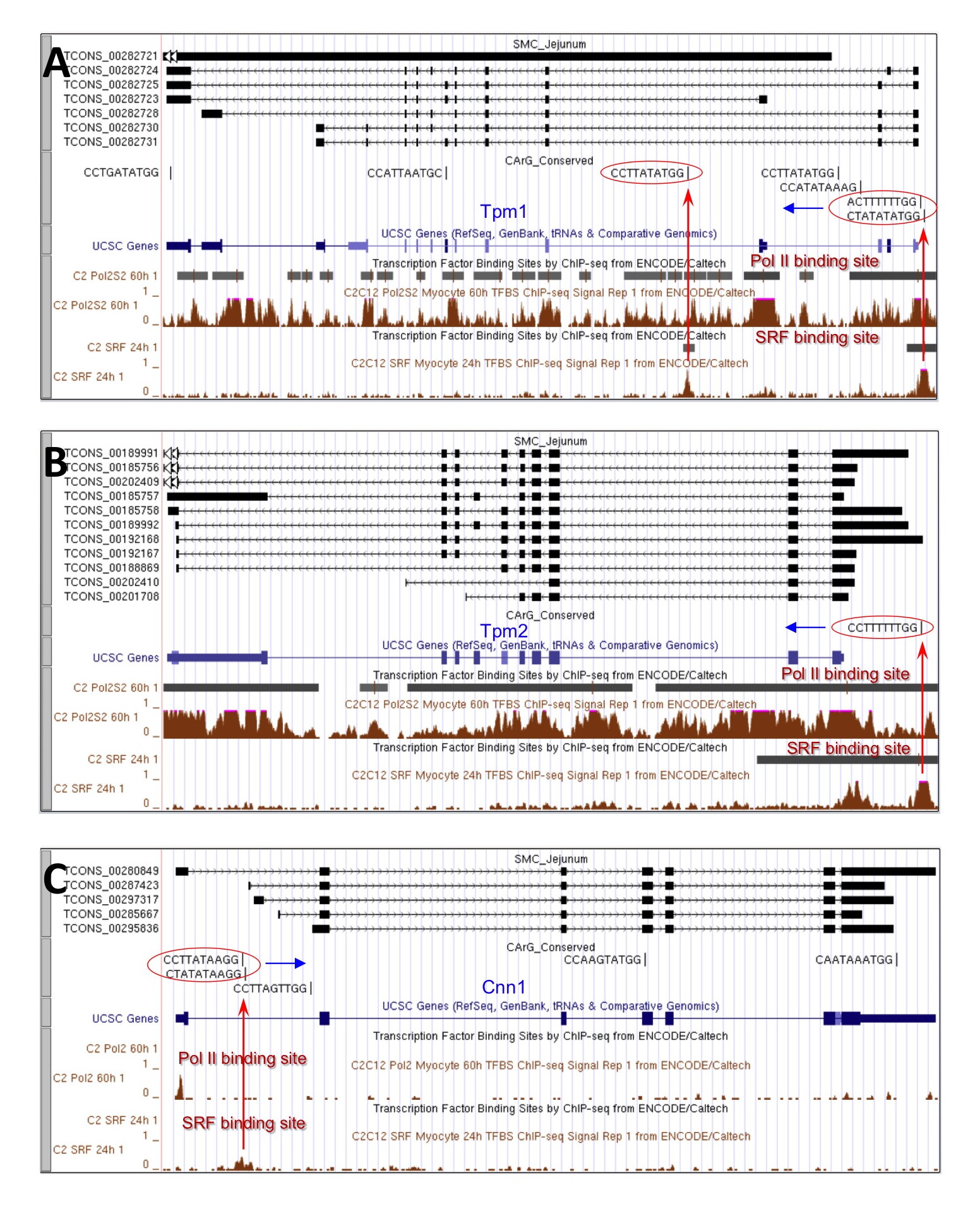

Supplement: S4 Fig — (A-C) A genomic map of Tpm1, Tpm2, and Cnn1 mRNA variants expressed in jejunal SMC showing conserved CArG boxes found within SRF binding sites from publically available data (C2C12). Binding sites of RNA Polymerase (Pol) II is shown in parallel with the variants and SRF binding sites. (TIF) [file pone.0133751.s005.tif]

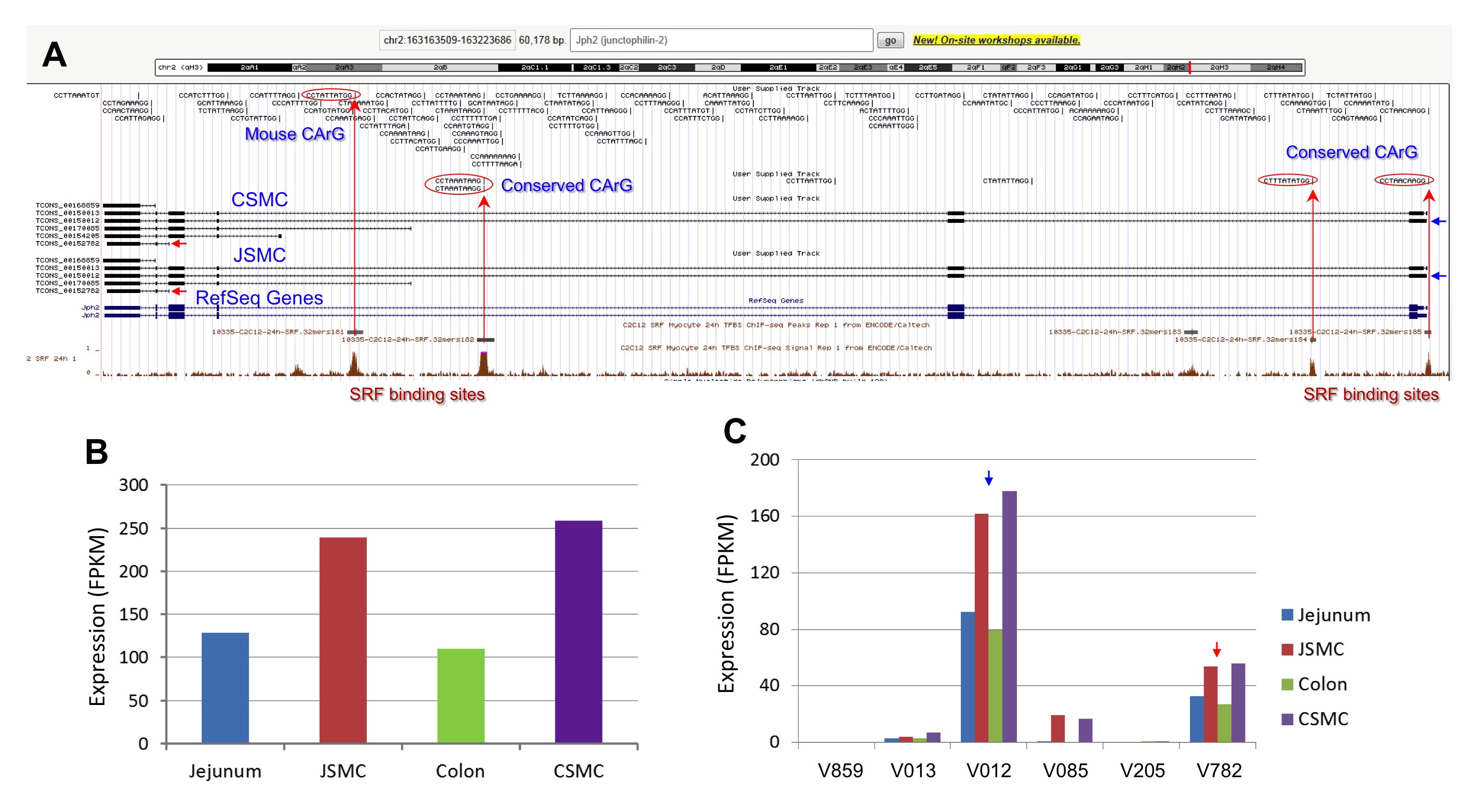

Supplement: S6 Fig — (A) A genomic map view of Jph2 variants expressed in JSMCs and CSMCs. Six Jph2 transcriptional variants are expressed in SMCs. There are four SRF binding sites on the promoter region, intron 1, and intron 2. Each SRF binding site contains one to two CArG boxes that are either mouse-specific or conserved between humans and mice. (B) Total expression levels (FPKM) of Jph2 in SMCs. (C) Expression levels (FPKM) of individual Jph2 variants in SMCs. (TIF) [file pone.0133751.s007.tif]

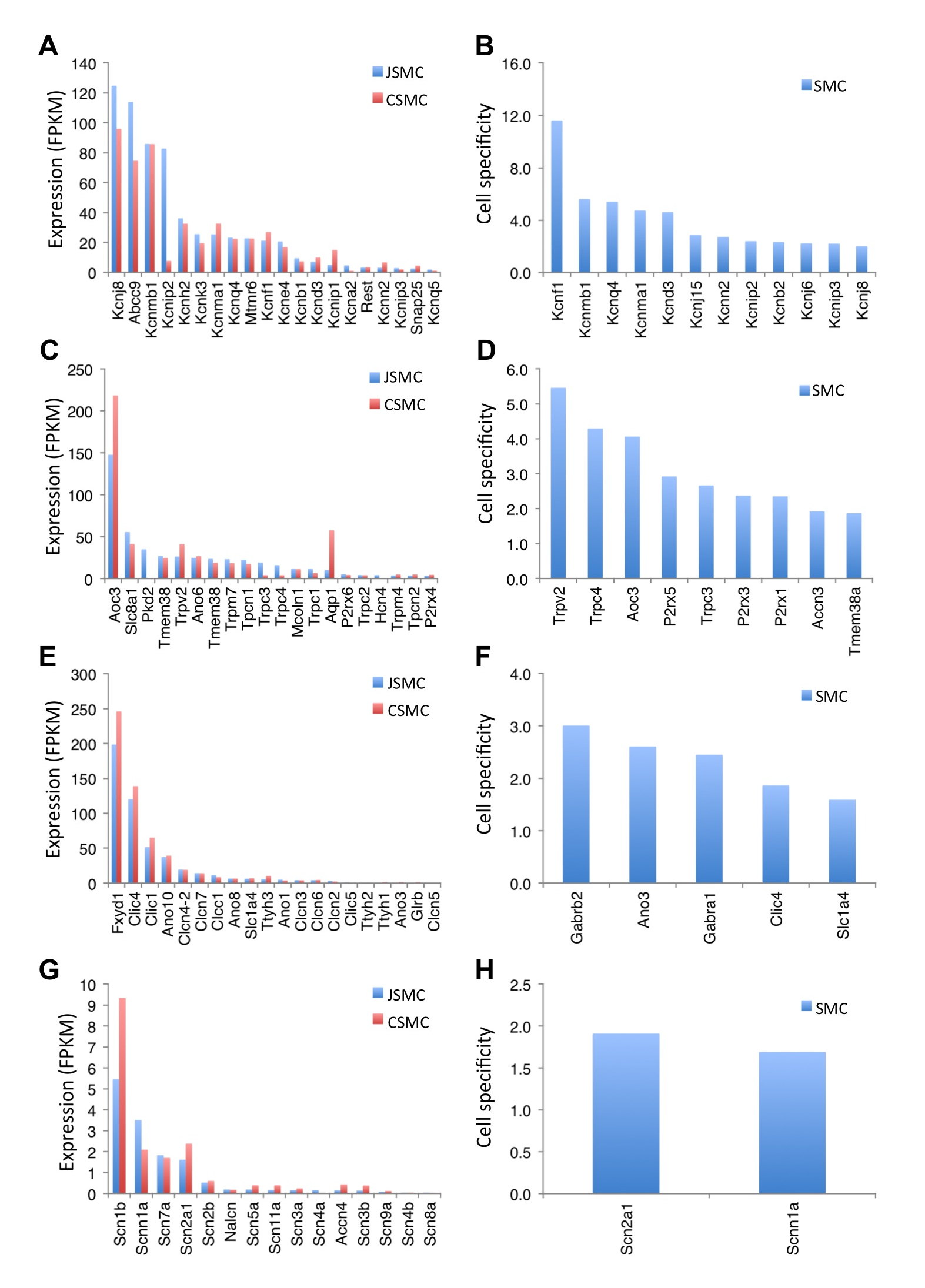

Supplement: S7 Fig — (A) Potassium channel isoforms enriched in JSMCs and CSMCs. (B) SMC-specific potassium channel isoforms. (C) Cation channel isoforms enriched in JSMCs and CSMCs. (D) SMC-specific cation channel isoforms. (E) Chloride channel isoforms enriched in JSMCs and CSMCs. (F) SMC-specific chloride channel isoforms. (G) Sodium channel isoforms enriched in JSMCs and CSMCs. (H) SMC-specific sodium channel isoforms. Cell specificity was determined by comparative analysis of gene expression profiles among SMCs, ICC, and PDGFRα+ cells. (TIF) [file pone.0133751.s008.tif]

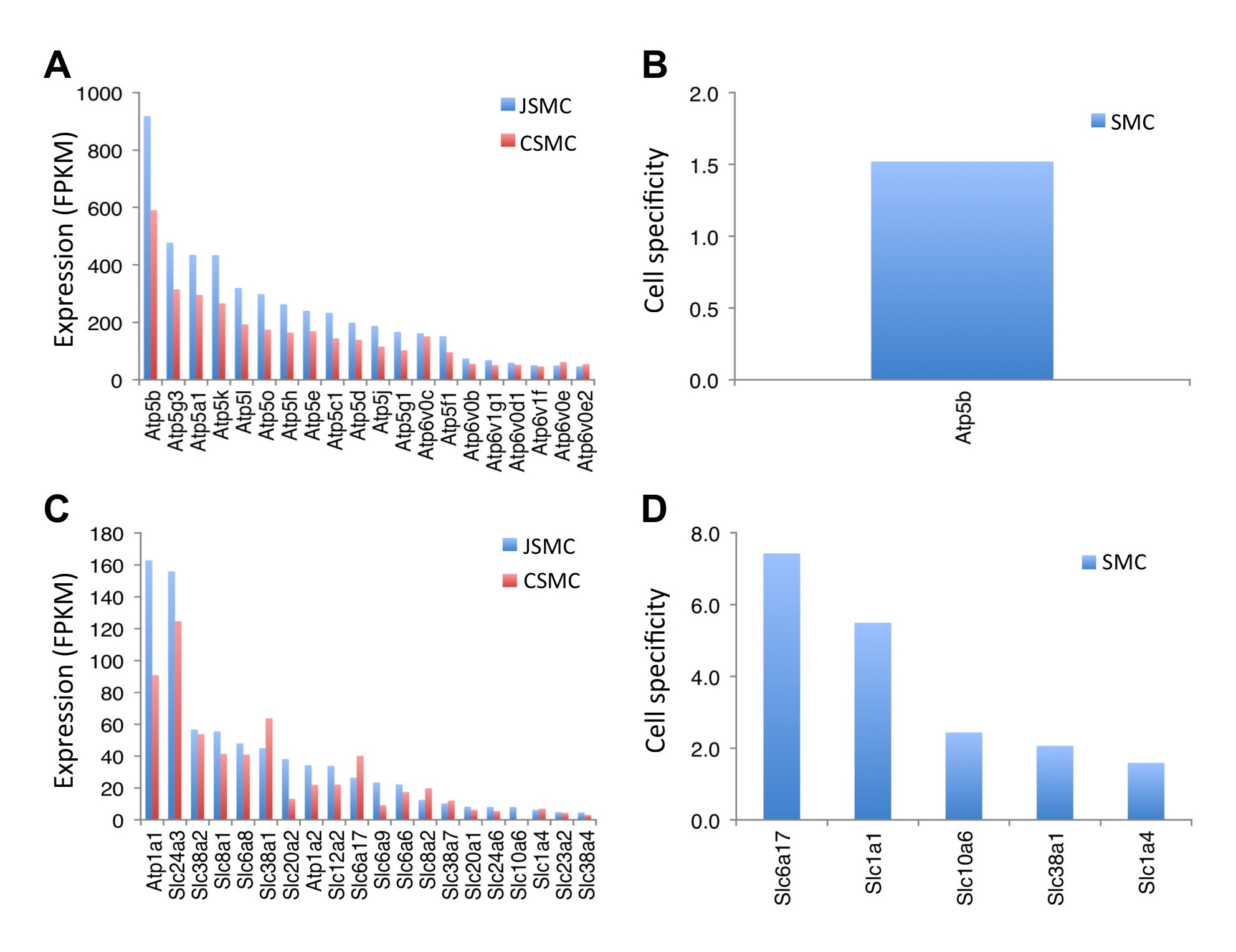

Supplement: S8 Fig — (A) Hydrogen transporter isoforms enriched in JSMCs and CSMCs. (B) SMC-specific hydrogen transporter isoform. (C) Sodium transporter isoforms enriched in JSMCs and CSMCs. (D) SMC-specific sodium transporter isoforms. Cell specificity was determined by comparative analysis of gene expression profiles among SMCs, ICC, and PDGFRα+ cells. (TIF) [file pone.0133751.s009.tif]
