# Supplementary material for: Smooth Muscle Cell Genome Browser: Enabling the Identification of Novel Serum Response Factor Target Genes
Source: PLoS One. 2015 Aug 4;10(8):e0133751. doi: 10.1371/journal.pone.0133751 (PMC4524680; doi:10.1371/journal.pone.0133751)
Supplement: S5 Fig — The open reading frame was identified for each transcriptional variant, and predicted amino acid sequences were aligned together. Seven transmembrane domains (TM1-7), a pore region, MR1-5, B30.2/SPRY1-3, FKBP1A, Repeat 1–4, CALM, and a residue E that is required for Ca2+ binding (Ca2+) are annotated along the amino acid sequences. Colors on the amino acid sequences designate start codons (green) and missing or inserted peptides (red) from differentially spliced or alternatively initiated exons. (DOCX) [file pone.0133751.s006.docx]

V497 MLVEATISIFHESIRHLDDHLLHYRPFNFYDPCFPMQEDEVVLQCIANIHKEQRKFCLAA 60

V367 MLVEATISIFHESIRHLDDHLLHYRPFNFYDPCFPMQEDEVVLQCIANIHKEQRKFCLAA 60

V672 --------------------MAEAGEGGEDEIQFLRTEDEVVLQCIANIHKEQRKFCLAA 40

V993 MLVEATISIFHESIRHLDDHLLHYRPFNFYDPCFPMQEDEVVLQCIANIHKEQRKFCLAA 60

V671 MLVEATISIFHESIRHLDDHLLHYRPFNFYDPCFPMQEDEVVLQCIANIHKEQRKFCLAA 60

V098 MLVEATISIFHESIRHLDDHLLHYRPFNFYDPCFPMQEDEVVLQCIANIHKEQRKFCLAA 60

V498 --------------------MAEAGEGGEDEIQFLRTEDEVVLQCIANIHKEQRKFCLAA 40

-

V497 EGLGNRLCFLEPTSEAKYIPPDLCVCNFVLEQSLSVRALQEMLANTVENGGEGAAQGGGH 120

V367 EGLGNRLCFLEPTSEAKYIPPDLCVCNFVLEQSLSVRALQEMLANTVENGGEGAAQGGGH 120

V672 EGLGNRLCFLEPTSEAKYIPPDLCVCNFVLEQSLSVRALQEMLANTVENGGEGAAQGGGH 100

V993 EGLGNRLCFLEPTSEAKYIPPDLCVCNFVLEQSLSVRALQEMLANTVENGGEGAAQGGGH 120

V671 EGLGNRLCFLEPTSEAKYIPPDLCVCNFVLEQSLSVRALQEMLANTVENGGEGAAQGGGH 120

V098 EGLGNRLCFLEPTSEAKYIPPDLCVCNFVLEQSLSVRALQEMLANTVENGGEGAAQGGGH 120

V498 EGLGNRLCFLEPTSEAKYIPPDLCVCNFVLEQSLSVRALQEMLANTVENGGEGAAQGGGH 100

------------------------- **MR1**---------------------------

V497 RTLLYGHAILLRHSFSGMYLTCLTTSRSQTDKLAFDVGLREHATGEACWWTIHPASKQRS 180

V367 RTLLYGHAILLRHSFSGMYLTCLTTSRSQTDKLAFDVGLREHATGEACWWTIHPASKQRS 180

V672 RTLLYGHAILLRHSFSGMYLTCLTTSRSQTDKLAFDVGLREHATGEACWWTIHPASKQRS 160

V993 RTLLYGHAILLRHSFSGMYLTCLTTSRSQTDKLAFDVGLREHATGEACWWTIHPASKQRS 180

V671 RTLLYGHAILLRHSFSGMYLTCLTTSRSQTDKLAFDVGLREHATGEACWWTIHPASKQRS 180

V098 RTLLYGHAILLRHSFSGMYLTCLTTSRSQTDKLAFDVGLREHATGEACWWTIHPASKQRS 180

V498 RTLLYGHAILLRHSFSGMYLTCLTTSRSQTDKLAFDVGLREHATGEACWWTIHPASKQRS 160

---------------------**MR2**---------------------- ------

V497 EGEKVRIGDDLILVSVSSERYLHLSISNGSIQVDASFMQTLWNVHPTCSGSSIEEGYLLG 240

V367 EGEKVRIGDDLILVSVSSERYLHLSISNGSIQVDASFMQTLWNVHPTCSGSSIEEGYLLG 240

V672 EGEKVRIGDDLILVSVSSERYLHLSISNGSIQVDASFMQTLWNVHPTCSGSSIEEGYLLG 220

V993 EGEKVRIGDDLILVSVSSERYLHLSISNGSIQVDASFMQTLWNVHPTCSGSSIEEGYLLG 240

V671 EGEKVRIGDDLILVSVSSERYLHLSISNGSIQVDASFMQTLWNVHPTCSGSSIEEGYLLG 240

V098 EGEKVRIGDDLILVSVSSERYLHLSISNGSIQVDASFMQTLWNVHPTCSGSSIEEGYLLG 240

V498 EGEKVRIGDDLILVSVSSERYLHLSISNGSIQVDASFMQTLWNVHPTCSGSSIEEGYLLG 220

-------------------**MR3**--------------------------- ------

V497 GHVVRLFHGHDECLTIPSTDQNDSQHRRVFYEAGGAGTRARSLWRVEPLRISWSGSNIRW 300

V367 GHVVRLFHGHDECLTIPSTDQNDSQHRRVFYEAGGAGTRARSLWRVEPLRISWSGSNIRW 300

V672 GHVVRLFHGHDECLTIPSTDQNDSQHRRVFYEAGGAGTRARSLWRVEPLRISWSGSNIRW 280

V993 GHVVRLFHGHDECLTIPSTDQNDSQHRRVFYEAGGAGTRARSLWRVEPLRISWSGSNIRW 300

V671 GHVVRLFHGHDECLTIPSTDQNDSQHRRVFYEAGGAGTRARSLWRVEPLRISWSGSNIRW 300

V098 GHVVRLFHGHDECLTIPSTDQNDSQHRRVFYEAGGAGTRARSLWRVEPLRISWSGSNIRW 300

V498 GHVVRLFHGHDECLTIPSTDQNDSQHRRVFYEAGGAGTRARSLWRVEPLRISWSGSNIRW 280

----------------------**MR4**----------------------------

V497 GQAFRLRHLTTGHYLALTEDQGLLLQDRGKSDTKSTAFSFRASKEIKEKLDSSHKRDMEG 360

V367 GQAFRLRHLTTGHYLALTEDQGLLLQDRGKSDTKSTAFSFRASKEIKEKLDSSHKRDMEG 360

V672 GQAFRLRHLTTGHYLALTEDQGLLLQDRGKSDTKSTAFSFRASKEIKEKLDSSHKRDMEG 340

V993 GQAFRLRHLTTGHYLALTEDQGLLLQDRGKSDTKSTAFSFRASKEIKEKLDSSHKRDMEG 360

V671 GQAFRLRHLTTGHYLALTEDQGLLLQDRGKSDTKSTAFSFRASKEIKEKLDSSHKRDMEG 360

V098 GQAFRLRHLTTGHYLALTEDQGLLLQDRGKSDTKSTAFSFRASKEIKEKLDSSHKRDMEG 360

V498 GQAFRLRHLTTGHYLALTEDQGLLLQDRGKSDTKSTAFSFRASKEIKEKLDSSHKRDMEG 340

---------------------------**MR5**----------------------------

V497 MGVPEIKYGDSVCFVQHVASGLWVTYKAQDAKTSRLGPLKRKVILHQEGHMDDGLTLQRC 420

V367 MGVPEIKYGDSVCFVQHVASGLWVTYKAQDAKTSRLGPLKRKVILHQEGHMDDGLTLQRC 420

V672 MGVPEIKYGDSVCFVQHVASGLWVTYKAQDAKTSRLGPLKRKVILHQEGHMDDGLTLQRC 400

V993 MGVPEIKYGDSVCFVQHVASGLWVTYKAQDAKTSRLGPLKRKVILHQEGHMDDGLTLQRC 420

V671 MGVPEIKYGDSVCFVQHVASGLWVTYKAQDAKTSRLGPLKRKVILHQEGHMDDGLTLQRC 420

V098 MGVPEIKYGDSVCFVQHVASGLWVTYKAQDAKTSRLGPLKRKVILHQEGHMDDGLTLQRC 420

V498 MGVPEIKYGDSVCFVQHVASGLWVTYKAQDAKTSRLGPLKRKVILHQEGHMDDGLTLQRC 400

V497 QQEESQAARIIRNTTALFSQFVSGNNRTTAPVALPTEEVLQTLQDLIAYFQPPEDEMQHE 480

V367 QQEESQAARIIRNTTALFSQFVSGNNRTTAPVALPTEEVLQTLQDLIAYFQPPEDEMQHE 480

V672 QQEESQAARIIRNTTALFSQFVSGNNRTTAPVALPTEEVLQTLQDLIAYFQPPEDEMQHE 460

V993 QQEESQAARIIRNTTALFSQFVSGNNRTTAPVALPTEEVLQTLQDLIAYFQPPEDEMQHE 480

V671 QQEESQAARIIRNTTALFSQFVSGNNRTTAPVALPTEEVLQTLQDLIAYFQPPEDEMQHE 480

V098 QQEESQAARIIRNTTALFSQFVSGNNRTTAPVALPTEEVLQTLQDLIAYFQPPEDEMQHE 480

V498 QQEESQAARIIRNTTALFSQFVSGNNRTTAPVALPTEEVLQTLQDLIAYFQPPEDEMQHE 460

V497 DKQNKLRSLKNRQNLFKEEGMLALVLNCIDRLNIYNSVAHFAGIVREESGMAWKEILNLL 540

V367 DKQNKLRSLKNRQNLFKEEGMLALVLNCIDRLNIYNSVAHFAGIVREESGMAWKEILNLL 540

V672 DKQNKLRSLKNRQNLFKEEGMLALVLNCIDRLNIYNSVAHFAGIVREESGMAWKEILNLL 520

V993 DKQNKLRSLKNRQNLFKEEGMLALVLNCIDRLNIYNSVAHFAGIVREESGMAWKEILNLL 540

V671 DKQNKLRSLKNRQNLFKEEGMLALVLNCIDRLNIYNSVAHFAGIVREESGMAWKEILNLL 540

V098 DKQNKLRSLKNRQNLFKEEGMLALVLNCIDRLNIYNSVAHFAGIVREESGMAWKEILNLL 540

V498 DKQNKLRSLKNRQNLFKEEGMLALVLNCIDRLNIYNSVAHFAGIVREESGMAWKEILNLL 520

V497 YKLLAALIRGNRNNCAQFSNNLDWLISKLDRLESSSGILEVLHCILIESPEALNLIAEGH 600

V367 YKLLAALIRGNRNNCAQFSNNLDWLISKLDRLESSSGILEVLHCILIESPEALNLIAEGH 600

V672 YKLLAALIRGNRNNCAQFSNNLDWLISKLDRLESSSGILEVLHCILIESPEALNLIAEGH 580

V993 YKLLAALIRGNRNNCAQFSNNLDWLISKLDRLESSSGILEVLHCILIESPEALNLIAEGH 600

V671 YKLLAALIRGNRNNCAQFSNNLDWLISKLDRLESSSGILEVLHCILIESPEALNLIAEGH 600

V098 YKLLAALIRGNRNNCAQFSNNLDWLISKLDRLESSSGILEVLHCILIESPEALNLIAEGH 600

V498 YKLLAALIRGNRNNCAQFSNNLDWLISKLDRLESSSGILEVLHCILIESPEALNLIAEGH 580

--------------------------------------------------------

V497 IKSIISLLDKHGRNHKVLDVLCSLCLCNGVAVRANQNLICDNLLPRRNLLLQTRLINDVT 660

V367 IKSIISLLDKHGRNHKVLDVLCSLCLCNGVAVRANQNLICDNLLPRRNLLLQTRLINDVT 660

V672 IKSIISLLDKHGRNHKVLDVLCSLCLCNGVAVRANQNLICDNLLPRRNLLLQTRLINDVT 640

V993 IKSIISLLDKHGRNHKVLDVLCSLCLCNGVAVRANQNLICDNLLPRRNLLLQTRLINDVT 660

V671 IKSIISLLDKHGRNHKVLDVLCSLCLCNGVAVRANQNLICDNLLPRRNLLLQTRLINDVT 660

V098 IKSIISLLDKHGRNHKVLDVLCSLCLCNGVAVRANQNLICDNLLPRRNLLLQTRLINDVT 660

V498 IKSIISLLDKHGRNHKVLDVLCSLCLCNGVAVRANQNLICDNLLPRRNLLLQTRLINDVT 640

----------------------------------------**B30.2/SPRY1**---------

V497 SIRPNIFLGVAEGSPQYKKWYFELIIDQVEPFLTAEPTHLRVGWASSSGYAPYPGGGEGW 720

V367 SIRPNIFLGVAEGSPQYKKWYFELIIDQVEPFLTAEPTHLRVGWASSSGYAPYPGGGEGW 720

V672 SIRPNIFLGVAEGSPQYKKWYFELIIDQVEPFLTAEPTHLRVGWASSSGYAPYPGGGEGW 700

V993 SIRPNIFLGVAEGSPQYKKWYFELIIDQVEPFLTAEPTHLRVGWASSSGYAPYPGGGEGW 720

V671 SIRPNIFLGVAEGSPQYKKWYFELIIDQVEPFLTAEPTHLRVGWASSSGYAPYPGGGEGW 720

V098 SIRPNIFLGVAEGSPQYKKWYFELIIDQVEPFLTAEPTHLRVGWASSSGYAPYPGGGEGW 720

V498 SIRPNIFLGVAEGSPQYKKWYFELIIDQVEPFLTAEPTHLRVGWASSSGYAPYPGGGEGW 700

------------------------------------------------------------

V497 GGNGVGDDLYSYGFDGLHLWSGRIPRAVASINQHLLKSDDVVSCCLDLGVPSISFRINGQ 780

V367 GGNGVGDDLYSYGFDGLHLWSGRIPRAVASINQHLLKSDDVVSCCLDLGVPSISFRINGQ 780

V672 GGNGVGDDLYSYGFDGLHLWSGRIPRAVASINQHLLKSDDVVSCCLDLGVPSISFRINGQ 760

V993 GGNGVGDDLYSYGFDGLHLWSGRIPRAVASINQHLLKSDDVVSCCLDLGVPSISFRINGQ 780

V671 GGNGVGDDLYSYGFDGLHLWSGRIPRAVASINQHLLKSDDVVSCCLDLGVPSISFRINGQ 780

V098 GGNGVGDDLYSYGFDGLHLWSGRIPRAVASINQHLLKSDDVVSCCLDLGVPSISFRINGQ 780

V498 GGNGVGDDLYSYGFDGLHLWSGRIPRAVASINQHLLKSDDVVSCCLDLGVPSISFRINGQ 760

------------------------------------

V497 PVQGMFENFNTDGLFFPVMSFSAGVKVRFLMGGRHGEFKFLPPSGYAPCYEALLPKEKMR 840

V367 PVQGMFENFNTDGLFFPVMSFSAGVKVRFLMGGRHGEFKFLPPSGYAPCYEALLPKEKMR 840

V672 PVQGMFENFNTDGLFFPVMSFSAGVKVRFLMGGRHGEFKFLPPSGYAPCYEALLPKEKMR 820

V993 PVQGMFENFNTDGLFFPVMSFSAGVKVRFLMGGRHGEFKFLPPSGYAPCYEALLPKEKMR 840

V671 PVQGMFENFNTDGLFFPVMSFSAGVKVRFLMGGRHGEFKFLPPSGYAPCYEALLPKEKMR 840

V098 PVQGMFENFNTDGLFFPVMSFSAGVKVRFLMGGRHGEFKFLPPSGYAPCYEALLPKEKMR 840

V498 PVQGMFENFNTDGLFFPVMSFSAGVKVRFLMGGRHGEFKFLPPSGYAPCYEALLPKEKMR 820

-----------------------------------------

V497 LEPVKEYKRDADGVRDLLGTTQFLSQASFIPCPIDTSQVVLPLHLEKIRDRLAENIHELW 900

V367 LEPVKEYKRDADGVRDLLGTTQFLSQASFIPCPIDTSQ---------------------- 877

V672 LEPVKEYKRDADGVRDLLGTTQFLSQASFIPCPIDTSQVVLPLHLEKIRDRLAENIHELW 880

V993 LEPVKEYKRDADGVRDLLGTTQFLSQASFIPCPIDTSQVVLPLHLEKIRDRLAENIHELW 900

V671 LEPVKEYKRDADGVRDLLGTTQFLSQASFIPCPIDTSQVVLPLHLEKIRDRLAENIHELW 900

V098 LEPVKEYKRDADGVRDLLGTTQFLSQASFIPCPIDTSQVVLPLHLEKIRDRLAENIHELW 900

V498 LEPVKEYKRDADGVRDLLGTTQFLSQASFIPCPIDTSQVVLPLHLEKIRDRLAENIHELW 880

--------------------**Repeat1**---------------------------------

V497 GMNKIELGWTYGKVRDDNKRQHPCLVEFSKLPETEKNYNLQMSTETLKTLLALGCHIAHV 960

V367 -------------VRDDNKRQHPCLVEFSKLPETEKNYNLQMSTETLKTLLALGCHIAHV 925

V672 GMNKIELGWTYGKVRDDNKRQHPCLVEFSKLPETEKNYNLQMSTETLKTLLALGCHIAHV 940

V993 GMNKIELGWTYGKVRDDNKRQHPCLVEFSKLPETEKNYNLQMSTETLKTLLALGCHIAHV 960

V671 GMNKIELGWTYGKVRDDNKRQHPCLVEFSKLPETEKNYNLQMSTETLKTLLALGCHIAHV 960

V098 GMNKIELGWTYGKVRDDNKRQHPCLVEFSKLPETEKNYNLQMSTETLKTLLALGCHIAHV 960

V498 GMNKIELGWTYGKVRDDNKRQHPCLVEFSKLPETEKNYNLQMSTETLKTLLALGCHIAHV 940

-----------------------------------------------

-------------

V497 NPAAEEDLKKVKLPKNYMMSNGYKPAPLDLSDVKLLPPQEILVDKLAENAHNVWAKDRIK 1020

V367 NPAAEEDLKKVKLPKNYMMSNGYKPAPLDLSDVKLLPPQEILVDKLAENAHNVWAKDRIK 985

V672 NPAAEEDLKKVKLPKNYMMSNGYKPAPLDLSDVKLLPPQEILVDKLAENAHNVWAKDRIK 1000

V993 NPAAEEDLKKVKLPKNYMMSNGYKPAPLDLSDVKLLPPQEILVDKLAENAHNVWAKDRIK 1020

V671 NPAAEEDLKKVKLPKNYMMSNGYKPAPLDLSDVKLLPPQEILVDKLAENAHNVWAKDRIK 1020

V098 NPAAEEDLKKVKLPKNYMMSNGYKPAPLDLSDVKLLPPQEILVDKLAENAHNVWAKDRIK 1020

V498 NPAAEEDLKKVKLPKNYMMSNGYKPAPLDLSDVKLLPPQEILVDKLAENAHNVWAKDRIK 1000

--------**Repeat2**---------------------------------------------

-------------------------------------------------

V497 QGWTYGIQQDLKNKRNPRLVPYALLDERTKKSNRDSLREAVRTFVGYGYNIEPSDQEL-A 1079

V367 QGWTYGIQQDLKNKRNPRLVPYALLDERTKKSNRDSLREAVRTFVGYGYNIEPSDQELVA 1045

V672 QGWTYGIQQDLKNKRNPRLVPYALLDERTKKSNRDSLREAVRTFVGYGYNIEPSDQELVA 1060

V993 QGWTYGIQQDLKNKRNPRLVPYALLDERTKKSNRDSLREAVRTFVGYGYNIEPSDQEL-A 1079

V671 QGWTYGIQQDLKNKRNPRLVPYALLDERTKKSNRDSLREAVRTFVGYGYNIEPSDQELVA 1080

V098 QGWTYGIQQDLKNKRNPRLVPYALLDERTKKSNRDSLREAVRTFVGYGYNIEPSDQEL-A 1079

V498 QGWTYGIQQDLKNKRNPRLVPYALLDERTKKSNRDSLREAVRTFVGYGYNIEPSDQEL-A 1059

---------

---------------------------------------------**B30.2/SPRY2**----

V497 DPTVEKVSIDKIRFFRVERSYAVKSGKWYFEFEVVTGGDMRVGWARPGCRPDIELGADDQ 1139

V367 DPTVEKVSIDKIRFFRVERSYAVKSGKWYFEFEVVTGGDMRVGWARPGCRPDIELGADDQ 1105

V672 DPTVEKVSIDKIRFFRVERSYAVKSGKWYFEFEVVTGGDMRVGWARPGCRPDIELGADDQ 1120

V993 DPTVEKVSIDKIRFFRVERSYAVKSGKWYFEFEVVTGGDMRVGWARPGCRPDIELGADDQ 1139

V671 DPTVEKVSIDKIRFFRVERSYAVKSGKWYFEFEVVTGGDMRVGWARPGCRPDIELGADDQ 1140

V098 DPTVEKVSIDKIRFFRVERSYAVKSGKWYFEFEVVTGGDMRVGWARPGCRPDIELGADDQ 1139

V498 DPTVEKVSIDKIRFFRVERSYAVKSGKWYFEFEVVTGGDMRVGWARPGCRPDIELGADDQ 1119

------------------------------------------------------------

V497 AFVFEGSRGQRWHQGSGYFGRTWQPGDVVGCMINLDDASMVFTLNGELLITNKGSELAFA 1199

V367 AFVFEGSRGQRWHQGSGYFGRTWQPGDVVGCMINLDDASMVFTLNGELLITNKGSELAFA 1165

V672 AFVFEGSRGQRWHQGSGYFGRTWQPGDVVGCMINLDDASMVFTLNGELLITNKGSELAFA 1180

V993 AFVFEGSRGQRWHQGSGYFGRTWQPGDVVGCMINLDDASMVFTLNGELLITNKGSELAFA 1199

V671 AFVFEGSRGQRWHQGSGYFGRTWQPGDVVGCMINLDDASMVFTLNGELLITNKGSELAFA 1200

V098 AFVFEGSRGQRWHQGSGYFGRTWQPGDVVGCMINLDDASMVFTLNGELLITNKGSELAFA 1199

V498 AFVFEGSRGQRWHQGSGYFGRTWQPGDVVGCMINLDDASMVFTLNGELLITNKGSELAFA 1179

-----------------------------

V497 DYEIENGFVPICSLGLSQIGRMNLGTDASTFKFYTMCGLQEGFEPFAVNMNRDVAVWFSK 1259

V367 DYEIENGFVPICSLGLSQIGRMNLGTDASTFKFYTMCGLQEGFEPFAVNMNRDVAVWFSK 1225

V672 DYEIENGFVPICSLGLSQIGRMNLGTDASTFKFYTMCGLQEGFEPFAVNMNRDVAVWFSK 1240

V993 DYEIENGFVPICSLGLSQIGRMNLGTDASTFKFYTMCGLQEGFEPFAVNMNRDVAVWFSK 1259

V671 DYEIENGFVPICSLGLSQIGRMNLGTDASTFKFYTMCGLQEGFEPFAVNMNRDVAVWFSK 1260

V098 DYEIENGFVPICSLGLSQIGRMNLGTDASTFKFYTMCGLQEGFEPFAVNMNRDVAVWFSK 1259

V498 DYEIENGFVPICSLGLSQIGRMNLGTDASTFKFYTMCGLQEGFEPFAVNMNRDVAVWFSK 1239

----------------------------------------------

V497 RLPTFVNVPKDHPHIEVVRIDGTMDSPPCLKVTHKTFGTQNSNANMIYCRLSMPVECHSS 1319

V367 RLPTFVNVPKDHPHIEVVRIDGTMDSPPCLKVTHKTFGTQNSNANMIYCRLSMPVECHSS 1285

V672 RLPTFVNVPKDHPHIEVVRIDGTMDSPPCLKVTHKTFGTQNSNANMIYCRLSMPVECHSS 1300

V993 RLPTFVNVPKDHPHIEVVRIDGTMDSPPCLKVTHKTFGTQNSNANMIYCRLSMPVECHSS 1319

V671 RLPTFVNVPKDHPHIEVVRIDGTMDSPPCLKVTHKTFGTQNSNANMIYCRLSMPVECHSS 1320

V098 RLPTFVNVPKDHPHIEVVRIDGTMDSPPCLKVTHKTFGTQNSNANMIYCRLSMPVECHSS 1319

V498 RLPTFVNVPKDHPHIEVVRIDGTMDSPPCLKVTHKTFGTQNSNANMIYCRLSMPVECHSS 1299

-------------------------------------------------**B30.2/SPRY3**

V497 FSHSPCLDSEAFQKRKQMQEILSHTTTQCYYAIRIFAGQDPSCVWVGWVTPDYHLYSEKF 1379

V367 FSHSPCLDSEAFQKRKQMQEILSHTTTQCYYAIRIFAGQDPSCVWVGWVTPDYHLYSEKF 1345

V672 FSHSPCLDSEAFQKRKQMQEILSHTTTQCYYAIRIFAGQDPSCVWVGWVTPDYHLYSEKF 1360

V993 FSHSPCLDSEAFQKRKQMQEILSHTTTQCYYAIRIFAGQDPSCVWVGWVTPDYHLYSEKF 1379

V671 FSHSPCLDSEAFQKRKQMQEILSHTTTQCYYAIRIFAGQDPSCVWVGWVTPDYHLYSEKF 1380

V098 FSHSPCLDSEAFQKRKQMQEILSHTTTQCYYAIRIFAGQDPSCVWVGWVTPDYHLYSEKF 1379

V498 FSHSPCLDSEAFQKRKQMQEILSHTTTQCYYAIRIFAGQDPSCVWVGWVTPDYHLYSEKF 1359

------------------------------------------------------------

V497 DLNKNCTVTVTLGDERGRVHESVKRSNCYMVWGGDIVASSQRSSRSNVDLEIGCLLDLAM 1439

V367 DLNKNCTVTVTLGDERGRVHESVKRSNCYMVWGGDIVASSQRSSRSNVDLEIGCLLDLAM 1405

V672 DLNKNCTVTVTLGDERGRVHESVKRSNCYMVWGGDIVASSQRSSRSNVDLEIGCLLDLAM 1420

V993 DLNKNCTVTVTLGDERGRVHESVKRSNCYMVWGGDIVASSQRSSRSNVDLEIGCLLDLAM 1439

V671 DLNKNCTVTVTLGDERGRVHESVKRSNCYMVWGGDIVASSQRSSRSNVDLEIGCLLDLAM 1440

V098 DLNKNCTVTVTLGDERGRVHESVKRSNCYMVWGGDIVASSQRSSRSNVDLEIGCLLDLAM 1439

V498 DLNKNCTVTVTLGDERGRVHESVKRSNCYMVWGGDIVASSQRSSRSNVDLEIGCLLDLAM 1419

-----------------------------------------------

V497 GMLSFSANGKELGTCYQVEPNTKVFPAVFLQPTSTSLFQFELGKLKNAMPLSAAIFKSEE 1499

V367 GMLSFSANGKELGTCYQVEPNTKVFPAVFLQPTSTSLFQFELGKLKNAMPLSAAIFKSEE 1465

V672 GMLSFSANGKELGTCYQVEPNTKVFPAVFLQPTSTSLFQFELGKLKNAMPLSAAIFKSEE 1480

V993 GMLSFSANGKELGTCYQVEPNTKVFPAVFLQPTSTSLFQFELGKLKNAMPLSAAIFKSEE 1499

V671 GMLSFSANGKELGTCYQVEPNTKVFPAVFLQPTSTSLFQFELGKLKNAMPLSAAIFKSEE 1500

V098 GMLSFSANGKELGTCYQVEPNTKVFPAVFLQPTSTSLFQFELGKLKNAMPLSAAIFKSEE 1499

V498 GMLSFSANGKELGTCYQVEPNTKVFPAVFLQPTSTSLFQFELGKLKNAMPLSAAIFKSEE 1479

V497 KNPTPQCPPRLDVQTIQPVLWSRMPSSFLKVETERVSERHGWVVQCLEPLQMMALHIPEE 1559

V367 KNPTPQCPPRLDVQTIQPVLWSRMPSSFLKVETERVSERHGWVVQCLEPLQMMALHIPEE 1525

V672 KNPTPQCPPRLDVQTIQPVLWSRMPSSFLKVETERVSERHGWVVQCLEPLQMMALHIPEE 1540

V993 KNPTPQCPPRLDVQTIQPVLWSRMPSSFLKVETERVSERHGWVVQCLEPLQMMALHIPEE 1559

V671 KNPTPQCPPRLDVQTIQPVLWSRMPSSFLKVETERVSERHGWVVQCLEPLQMMALHIPEE 1560

V098 KNPTPQCPPRLDVQTIQPVLWSRMPSSFLKVETERVSERHGWVVQCLEPLQMMALHIPEE 1559

V498 KNPTPQCPPRLDVQTIQPVLWSRMPSSFLKVETERVSERHGWVVQCLEPLQMMALHIPEE 1539

V497 NRCVDILELCEQEDLMQFHYHTLRLYSAVCALGNSRVASALCSHVDLSQLFYAIDNKYLP 1619

V367 NRCVDILELCEQEDLMQFHYHTLRLYSAVCALGNSRVASALCSHVDLSQLFYAIDNKYLP 1585

V672 NRCVDILELCEQEDLMQFHYHTLRLYSAVCALGNSRVASALCSHVDLSQLFYAIDNKYLP 1600

V993 NRCVDILELCEQEDLMQFHYHTLRLYSAVCALGNSRVASALCSHVDLSQLFYAIDNKYLP 1619

V671 NRCVDILELCEQEDLMQFHYHTLRLYSAVCALGNSRVASALCSHVDLSQLFYAIDNKYLP 1620

V098 NRCVDILELCEQEDLMQFHYHTLRLYSAVCALGNSRVASALCSHVDLSQLFYAIDNKYLP 1619

V498 NRCVDILELCEQEDLMQFHYHTLRLYSAVCALGNSRVASALCSHVDLSQLFYAIDNKYLP 1599

V497 GLLRSGFYDLLISIHLANAKERKLMMKNEYIIPITSATRNIRLYPDESKRHGLPGVGLRT 1679

V367 GLLRSGFYDLLISIHLANAKERKLMMKNEYIIPITSATRNIRLYPDESKRHGLPGVGLRT 1645

V672 GLLRSGFYDLLISIHLANAKERKLMMKNEYIIPITSATRNIRLYPDESKRHGLPGVGLRT 1660

V993 GLLRSGFYDLLISIHLANAKERKLMMKNEYIIPITSATRNIRLYPDESKRHGLPGVGLRT 1679

V671 GLLRSGFYDLLISIHLANAKERKLMMKNEYIIPITSATRNIRLYPDESKRHGLPGVGLRT 1680

V098 GLLRSGFYDLLISIHLANAKERKLMMKNEYIIPITSATRNIRLYPDESKRHGLPGVGLRT 1679

V498 GLLRSGFYDLLISIHLANAKERKLMMKNEYIIPITSATRNIRLYPDESKRHGLPGVGLRT 1659

V497 CLKPGFRFSTPCFVVTSEDHQKQSPEIPLQILKTKALSMLTEAVHCSGAHIRDPVGGSVE 1739

V367 CLKPGFRFSTPCFVVTSEDHQKQSPEIPLQILKTKALSMLTEAVHCSGAHIRDPVGGSVE 1705

V672 CLKPGFRFSTPCFVVTSEDHQKQSPEIPLQILKTKALSMLTEAVHCSGAHIRDPVGGSVE 1720

V993 CLKPGFRFSTPCFVVTSEDHQKQSPEIPLQILKTKALSMLTEAVHCSGAHIRDPVGGSVE 1739

V671 CLKPGFRFSTPCFVVTSEDHQKQSPEIPLQILKTKALSMLTEAVHCSGAHIRDPVGGSVE 1740

V098 CLKPGFRFSTPCFVVTSEDHQKQSPEIPLQILKTKALSMLTEAVHCSGAHIRDPVGGSVE 1739

V498 CLKPGFRFSTPCFVVTSEDHQKQSPEIPLQILKTKALSMLTEAVHCSGAHIRDPVGGSVE 1719

V497 FQFVPVLKLIGTLLVMGVFDDDDVRQILLLIDPSVFGEHSGETEEGVEKEVTHAEEKAVE 1799

V367 FQFVPVLKLIGTLLVMGVFDDDDVRQILLLIDPSVFGEHSGETEEGVEKEVTHAEEKAVE 1765

V672 FQFVPVLKLIGTLLVMGVFDDDDVRQILLLIDPSVFGEHSGETEEGVEKEVTHAEEKAVE 1780

V993 FQFVPVLKLIGTLLVMGVFDDDDVRQILLLIDPSVFGEHSGETEEGVEKEVTHAEEKAVE 1799

V671 FQFVPVLKLIGTLLVMGVFDDDDVRQILLLIDPSVFGEHSGETEEGVEKEVTHAEEKAVE 1800

V098 FQFVPVLKLIGTLLVMGVFDDDDVRQILLLIDPSVFGEHSGETEEGVEKEVTHAEEKAVE 1799

V498 FQFVPVLKLIGTLLVMGVFDDDDVRQILLLIDPSVFGEHSGETEEGVEKEVTHAEEKAVE 1779

V497 AGEKACKEAPVKGLLQTRLPESVKLQMCELLSYLCDCELQHRVEAIVAFGDIYVSKLQAN 1859

V367 AGEKACKEAPVKGLLQTRLPESVKLQMCELLSYLCDCELQHRVEAIVAFGDIYVSKLQAN 1825

V672 AGEKACKEAPVKGLLQTRLPESVKLQMCELLSYLCDCELQHRVEAIVAFGDIYVSKLQAN 1840

V993 AGEKACKEAPVKGLLQTRLPESVKLQMCELLSYLCDCELQHRVEAIVAFGDIYVSKLQAN 1859

V671 AGEKACKEAPVKGLLQTRLPESVKLQMCELLSYLCDCELQHRVEAIVAFGDIYVSKLQAN 1860

V098 AGEKACKEAPVKGLLQTRLPESVKLQMCELLSYLCDCELQHRVEAIVAFGDIYVSKLQAN 1859

V498 AGEKACKEAPVKGLLQTRLPESVKLQMCELLSYLCDCELQHRVEAIVAFGDIYVSKLQAN 1839

V497 QKFRYNELMQALNMSAALTARKTREFRSPPQEQINMLLNFHLGENCPCPEEIREELYDFH 1919

V367 QKFRYNELMQALNMSAALTARKTREFRSPPQEQINMLLNFHLGENCPCPEEIREELYDFH 1885

V672 QKFRYNELMQALNMSAALTARKTREFRSPPQEQINMLLNFHLGENCPCPEEIREELYDFH 1900

V366 -------------MSAALTARKTREFRSPPQEQINMLLNFHLGENCPCPEEIREELYDFH 47

V993 QKFRYNELMQALNMSAALTARKTREFRSPPQEQINMLLNFHLGENCPCPEEIREELYDFH 1919

V671 QKFRYNELMQALNMSAALTARKTREFRSPPQEQINMLLNFHLGENCPCPEEIREELYDFH 1920

V098 QKFRYNELMQALNMSAALTARKTREFRSPPQEQINMLLNFHLGENCPCPEEIREELYDFH 1919

V498 QKFRYNELMQALNMSAALTARKTREFRSPPQEQINMLLNFHLGENCPCPEEIREELYDFH 1899

V497 EDLLVHCGVPLEEEEEEEEDTSWTGKLCALVYKIKGPPKPEKEQPTEEEKPYPTTLKELV 1979

V367 EDLLVHCGVPLEEEEEEEEDTSWTGKLCALVYKIKGPPKPEKEQPTEEEKPYPTTLKELV 1945

V672 EDLLVHCGVPLEEEEEEEEDTSWTGKLCALVYKIKGPPKPEKEQPTEEEKPYPTTLKELV 1960

V366 EDLLVHCGVPLEEEEEEEEDTSWTGKLCALVYKIKGPPKPEKEQPTEEEKPYPTTLKELV 107

V993 EDLLVHCGVPLEEEEEEEEDTSWTGKLCALVYKIKGPPKPEKEQPTEEEKPYPTTLKELV 1979

V671 EDLLVHCGVPLEEEEEEEEDTSWTGKLCALVYKIKGPPKPEKEQPTEEEKPYPTTLKELV 1980

V098 EDLLVHCGVPLEEEEEEEEDTSWTGKLCALVYKIKGPPKPEKEQPTEEEKPYPTTLKELV 1979

V498 EDLLVHCGVPLEEEEEEEEDTSWTGKLCALVYKIKGPPKPEKEQPTEEEKPYPTTLKELV 1959

V497 SQTMIRWAQENQIQDAELVRMMFNLLRRQYDSIGELLQALRKTYTISQASVNDTINLLAA 2039

V367 SQTMIRWAQENQIQDAELVRMMFNLLRRQYDSIGELLQALRKTYTISQASVNDTINLLAA 2005

V672 SQTMIRWAQENQIQDAELVRMMFNLLRRQYDSIGELLQALRKTYTISQASVNDTINLLAA 2020

V366 SQTMIRWAQENQIQDAELVRMMFNLLRRQYDSIGELLQALRKTYTISQASVNDTINLLAA 167

V993 SQTMIRWAQENQIQDAELVRMMFNLLRRQYDSIGELLQALRKTYTISQASVNDTINLLAA 2039

V671 SQTMIRWAQENQIQDAELVRMMFNLLRRQYDSIGELLQALRKTYTISQASVNDTINLLAA 2040

V098 SQTMIRWAQENQIQDAELVRMMFNLLRRQYDSIGELLQALRKTYTISQASVNDTINLLAA 2039

V498 SQTMIRWAQENQIQDAELVRMMFNLLRRQYDSIGELLQALRKTYTISQASVNDTINLLAA 2019

V497 LGQIRSLLSVRMGREEELLMINGLGDIMNNKVFYQHPNLMRVLGMHETVMEVMVNVLGTE 2099

V367 LGQIRSLLSVRMGREEELLMINGLGDIMNNKVFYQHPNLMRVLGMHETVMEVMVNVLGTE 2065

V672 LGQIRSLLSVRMGREEELLMINGLGDIMNNKVFYQHPNLMRVLGMHETVMEVMVNVLGTE 2080

V366 LGQIRSLLSVRMGREEELLMINGLGDIMNNKVFYQHPNLMRVLGMHETVMEVMVNVLGTE 227

V993 LGQIRSLLSVRMGREEELLMINGLGDIMNNKVFYQHPNLMRVLGMHETVMEVMVNVLGTE 2099

V671 LGQIRSLLSVRMGREEELLMINGLGDIMNNKVFYQHPNLMRVLGMHETVMEVMVNVLGTE 2100

V098 LGQIRSLLSVRMGREEELLMINGLGDIMNNKVFYQHPNLMRVLGMHETVMEVMVNVLGTE 2099

V498 LGQIRSLLSVRMGREEELLMINGLGDIMNNKVFYQHPNLMRVLGMHETVMEVMVNVLGTE 2079

V497 KSQIAFPKMVASCCRFLCYFCRISRQNQKAMFEHLSYLLENSSVGLASPSMRGSTPLDVA 2159

V367 KSQIAFPKMVASCCRFLCYFCRISRQNQKAMFEHLSYLLENSSVGLGRNLLGRLWPSELP 2125

V672 KSQIAFPKMVASCCRFLCYFCRISRQNQKAMFEHLSYLLENSSVGLASPSMRGSTPLDVA 2140

V366 KSQIAFPKMVASCCRFLCYFCRISRQNQKAMFEHLSYLLENSSVGLASPSMRGSTPLDVA 287

V993 KSQIAFPKMVASCCRFLCYFCRISRQNQKAMFEHLSYLLENSSVGLASPSMRGSTPLDVA 2159

V671 KSQIAFPKMVASCCRFLCYFCRISRQNQKAMFEHLSYLLENSSVGLASPSMRGSTPLDVA 2160

V098 KSQIAFPKMVASCCRFLCYFCRISRQNQKAMFEHLSYLLENSSVGLASPSMRGSTPLDVA 2159

V498 KSQIAFPKMVASCCRFLCYFCRISRQNQKAMFEHLSYLLENSSVGLASPSMRGSTPLDVA 2139

V497 ASSVMDNNELALGLEEPDLEKVVTYLAGCGLQSCPMLLARGYPDVGWNPIEGERYLSFLR 2219

V367 HASGQRLP---------------------------------------------------- 2133

V672 ASSVMDNNELALGLEEPDLEKVVTYLAGCGLQSCPMLLARGYPDVGWNPIEGERYLSFLR 2200

V366 ASSVMDNNELALGLEEPDLEKVVTYLAGCGLQSCPMLLARGYPDVGWNPIEGERYLSFLR 347

V993 ASSVMDNNELALGLEEPDLEKVVTYLAGCGLQSCPMLLARGYPDVGWNPIEGERYLSFLR 2219

V671 ASSVMDNNELALGLEEPDLEKVVTYLAGCGLQSCPMLLARGYPDVGWNPIEGERYLSFLR 2220

V098 ASSVMDNNELALGLEEPDLEKVVTYLAGCGLQSCPMLLARGYPDVGWNPIEGERYLSFLR 2219

V498 ASSVMDNNELALGLEEPDLEKVVTYLAGCGLQSCPMLLARGYPDVGWNPIEGERYLSFLR 2199

V497 FAVFVNSESVEENASVVVKLLIRRPECFGPALRGEGGNGLLAAMQGAIKISENPALDLPS 2279

V672 FAVFVNSESVEENASVVVKLLIRRPECFGPALRGEGGNGLLAAMQGAIKISENPALDLPS 2260

V366 FAVFVNSESVEENASVVVKLLIRRPECFGPALRGEGGNGLLAAMQGAIKISENPALDLPS 407

V993 FAVFVNSESVEENASVVVKLLIRRPECFGPALRGEGGNGLLAAMQGAIKISENPALDLPS 2279

V671 FAVFVNSESVEENASVVVKLLIRRPECFGPALRGEGGNGLLAAMQGAIKISENPALDLPS 2280

V098 FAVFVNSESVEENASVVVKLLIRRPECFGPALRGEGGNGLLAAMQGAIKISENPALDLPS 2279

V498 FAVFVNSESVEENASVVVKLLIRRPECFGPALRGEGGNGLLAAMQGAIKISENPALDLPS 2259

V497 QGYKTEVTQDDGEEEEIVHMGNAIMSFYSALIDLLGRCAPEMHLIQTGKGEAIRIRSILR 2339

V672 QGYKTEVTQDDGEEEEIVHMGNAIMSFYSALIDLLGRCAPEMHLIQTGKGEAIRIRSILR 2320

V366 QGYKTEVTQDDGEEEEIVHMGNAIMSFYSALIDLLGRCAPEMHLIQTGKGEAIRIRSILR 467

V993 QGYKTEVTQDDGEEEEIVHMGNAIMSFYSALIDLLGRCAPEMHLIQTGKGEAIRIRSILR 2339

V671 QGYKTEVTQDDGEEEEIVHMGNAIMSFYSALIDLLGRCAPEMHLIQTGKGEAIRIRSILR 2340

V098 QGYKTEVTQDDGEEEEIVHMGNAIMSFYSALIDLLGRCAPEMHLIQTGKGEAIRIRSILR 2339

V498 QGYKTEVTQDDGEEEEIVHMGNAIMSFYSALIDLLGRCAPEMHLIQTGKGEAIRIRSILR 2319

----**FKBP1A**----

V497 SLVPTEDLVGIISIPLKLPSLNKDGSVSEPDMAANFCPDHKAPMVLFLDRVYGIKDQTFL 2399

V672 SLVPTEDLVGIISIPLKLPSLNKDGSVSEPDMAANFCPDHKAPMVLFLDRVYGIKDQTFL 2380

V366 SLVPTEDLVGIISIPLKLPSLNKDGSVSEPDMAANFCPDHKAPMVLFLDRVYGIKDQTFL 527

V993 SLVPTEDLVGIISIPLKLPSLNKDGSVSEPDMAANFCPDHKAPMVLFLDRVYGIKDQTFL 2399

V671 SLVPTEDLVGIISIPLKLPSLNKDGSVSEPDMAANFCPDHKAPMVLFLDRVYGIKDQTFL 2400

V098 SLVPTEDLVGIISIPLKLPSLNKDGSVSEPDMAANFCPDHKAPMVLFLDRVYGIKDQTFL 2399

V498 SLVPTEDLVGIISIPLKLPSLNKDGSVSEPDMAANFCPDHKAPMVLFLDRVYGIKDQTFL 2379

V497 LHLLEVGFLPDLRASASLDTVSLSTTEAALALNRYLCSAVLPLLTRCAPLFSGTEHCTSL 2459

V672 LHLLEVGFLPDLRASASLDTVSLSTTEAALALNRYLCSAVLPLLTRCAPLFSGTEHCTSL 2440

V366 LHLLEVGFLPDLRASASLDTVSLSTTEAALALNRYLCSAVLPLLTRCAPLFSGTEHCTSL 587

V993 LHLLEVGFLPDLRASASLDTVSLSTTEAALALNRYLCSAVLPLLTRCAPLFSGTEHCTSL 2459

V671 LHLLEVGFLPDLRASASLDTVSLSTTEAALALNRYLCSAVLPLLTRCAPLFSGTEHCTSL 2460

V098 LHLLEVGFLPDLRASASLDTVSLSTTEAALALNRYLCSAVLPLLTRCAPLFSGTEHCTSL 2459

V498 LHLLEVGFLPDLRASASLDTVSLSTTEAALALNRYLCSAVLPLLTRCAPLFSGTEHCTSL 2439

V497 IDSTLQTIYRLSKGRSLTKAQRDTIEECLLAICNHLRPSMLQQLLRRLVFDVPQLSEYCK 2519

V672 IDSTLQTIYRLSKGRSLTKAQRDTIEECLLAICNHLRPSMLQQLLRRLVFDVPQLSEYCK 2500

V366 IDSTLQTIYRLSKGRSLTKAQRDTIEECLLAICNHLRPSMLQQLLRRLVFDVPQLSEYCK 647

V993 IDSTLQTIYRLSKGRSLTKAQRDTIEECLLAICNHLRPSMLQQLLRRLVFDVPQLSEYCK 2519

V671 IDSTLQTIYRLSKGRSLTKAQRDTIEECLLAICNHLRPSMLQQLLRRLVFDVPQLSEYCK 2520

V098 IDSTLQTIYRLSKGRSLTKAQRDTIEECLLAICNHLRPSMLQQLLRRLVFDVPQLSEYCK 2519

V498 IDSTLQTIYRLSKGRSLTKAQRDTIEECLLAICNHLRPSMLQQLLRRLVFDVPQLSEYCK 2499

V497 MPLKLLTNHYEQCWKYYCLPSGWGSYGLAVEEELHLTEKLFWGIFDSLSHKKYDLDLFRM 2579

V672 MPLKLLTNHYEQCWKYYCLPSGWGSYGLAVEEELHLTEKLFWGIFDSLSHKKYDLDLFRM 2560

V366 MPLKLLTNHYEQCWKYYCLPSGWGSYGLAVEEELHLTEKLFWGIFDSLSHKKYDLDLFRM 707

V993 MPLKLLTNHYEQCWKYYCLPSGWGSYGLAVEEELHLTEKLFWGIFDSLSHKKYDLDLFRM 2579

V671 MPLKLLTNHYEQCWKYYCLPSGWGSYGLAVEEELHLTEKLFWGIFDSLSHKKYDLDLFRM 2580

V098 MPLKLLTNHYEQCWKYYCLPSGWGSYGLAVEEELHLTEKLFWGIFDSLSHKKYDLDLFRM 2579

V498 MPLKLLTNHYEQCWKYYCLPSGWGSYGLAVEEELHLTEKLFWGIFDSLSHKKYDLDLFRM 2559

---------------------------------

V497 ALPCLSAIAGALPPDYLDTRITATLEKQVSVDADGNFDPKPINTMNFSLPEKLEYIVTKY 2639

V672 ALPCLSAIAGALPPDYLDTRITATLEKQVSVDADGNFDPKPINTMNFSLPEKLEYIVTKY 2620

V366 ALPCLSAIAGALPPDYLDTRITATLEKQVSVDADGNFDPKPINTMNFSLPEKLEYIVTKY 767

V993 ALPCLSAIAGALPPDYLDTRITATLEKQVSVDADGNFDPKPINTMNFSLPEKLEYIVTKY 2639

V671 ALPCLSAIAGALPPDYLDTRITATLEKQVSVDADGNFDPKPINTMNFSLPEKLEYIVTKY 2640

V098 ALPCLSAIAGALPPDYLDTRITATLEKQVSVDADGNFDPKPINTMNFSLPEKLEYIVTKY 2639

V498 ALPCLSAIAGALPPDYLDTRITATLEKQVSVDADGNFDPKPINTMNFSLPEKLEYIVTKY 2619

-----------------------**Repeat3**------------------------------

V497 AEHSHDKWACDKSHSGWKYGISLDENVKTHPLIRPFKTLTEKEKEIYRWPARESLKTMLA 2699

V672 AEHSHDKWACDKSHSGWKYGISLDENVKTHPLIRPFKTLTEKEKEIYRWPARESLKTMLA 2680

V366 AEHSHDKWACDKSHSGWKYGISLDENVKTHPLIRPFKTLTEKEKEIYRWPARESLKTMLA 827

V993 AEHSHDKWACDKSHSGWKYGISLDENVKTHPLIRPFKTLTEKEKEIYRWPARESLKTMLA 2699

V671 AEHSHDKWACDKSHSGWKYGISLDENVKTHPLIRPFKTLTEKEKEIYRWPARESLKTMLA 2700

V098 AEHSHDKWACDKSHSGWKYGISLDENVKTHPLIRPFKTLTEKEKEIYRWPARESLKTMLA 2699

V498 AEHSHDKWACDKSHSGWKYGISLDENVKTHPLIRPFKTLTEKEKEIYRWPARESLKTMLA 2679

----------------------------------

--------------------------

V497 VGWTVERTKEGEALVQQRENEKLRCVSQTNQGNSYSPAPLDLSNVVLSRELQGMVEVVAE 2759

V672 VGWTVERTKEGEALVQQRENEKLRCVSQTNQGNSYSPAPLDLSNVVLSRELQGMVEVVAE 2740

V366 VGWTVERTKEGEALVQQRENEKLRCVSQTNQGNSYSPAPLDLSNVVLSRELQGMVEVVAE 887

V993 VGWTVERTKEGEALVQQRENEKLRCVSQTNQGNSYSPAPLDLSNVVLSRELQGMVEVVAE 2759

V671 VGWTVERTKEGEALVQQRENEKLRCVSQTNQGNSYSPAPLDLSNVVLSRELQGMVEVVAE 2760

V098 VGWTVERTKEGEALVQQRENEKLRCVSQTNQGNSYSPAPLDLSNVVLSRELQGMVEVVAE 2759

V498 VGWTVERTKEGEALVQQRENEKLRCVSQTNQGNSYSPAPLDLSNVVLSRELQGMVEVVAE 2739

-----------------------**Repeat4**------------------------------

V497 NYHNIWAKKKKLELESKGGGSHPLLVPYDTLTAKEKFRDREKAQDLFKFLQVNGILVSRG 2819

V672 NYHNIWAKKKKLELESKGGGSHPLLVPYDTLTAKEKFRDREKAQDLFKFLQVNGILVSRG 2800

V366 NYHNIWAKKKKLELESKGGGSHPLLVPYDTLTAKEKFRDREKAQDLFKFLQVNGILVSRG 947

V993 NYHNIWAKKKKLELESKGGGSHPLLVPYDTLTAKEKFRDREKAQDLFKFLQVNGILVSRG 2819

V671 NYHNIWAKKKKLELESKGGGSHPLLVPYDTLTAKEKFRDREKAQDLFKFLQVNGILVSRG 2820

V098 NYHNIWAKKKKLELESKGGGSHPLLVPYDTLTAKEKFRDREKAQDLFKFLQVNGILVSRG 2819

V498 NYHNIWAKKKKLELESKGGGSHPLLVPYDTLTAKEKFRDREKAQDLFKFLQVNGILVSRG 2799

-------------------

V497 MKDLELDASSMEKRFAYKFLKKILKYVDAAQEFIAHLEAIVSSGKTEKSPHDQEIKFFAK 2879

V672 MKDLELDASSMEKRFAYKFLKKILKYVDAAQEFIAHLEAIVSSGKTEKSPHDQEIKFFAK 2860

V366 MKDLELDASSMEKRFAYKFLKKILKYVDAAQEFIAHLEAIVSSGKTEKSPHDQEIKFFAK 1007

V993 MKDLELDASSMEKRFAYKFLKKILKYVDAAQEFIAHLEAIVSSGKTEKSPHDQEIKFFAK 2879

V671 MKDLELDASSMEKRFAYKFLKKILKYVDAAQEFIAHLEAIVSSGKTEKSPHDQEIKFFAK 2880

V098 MKDLELDASSMEKRFAYKFLKKILKYVDAAQEFIAHLEAIVSSGKTEKSPHDQEIKFFAK 2879

V498 MKDLELDASSMEKRFAYKFLKKILKYVDAAQEFIAHLEAIVSSGKTEKSPHDQEIKFFAK 2859

V497 VLLPLVDQYFTNHRLYFLSSPLKPLSSSGYASHKEKEMVASL-FCKLAALVRHRIS-LFG 2937

V672 VLLPLVDQYFTNHRLYFLSSPLKPLSSSGYASHKEKEMVASL-FCKLAALVRHRIS-LFG 2918

V366 VLLPLVDQYFTNHRLYFLSSPLKPLSSSGYASHKEKEMVASL-FCKLAALVRHRIS-LFG 1065

V993 VLLPLVDQYFTNHRLYFLSSPLKPLSSSGYASHKEKEMVASL-FCKLAALVRHRIS-LFG 2937

V671 VLLPLVDQYFTNHRLYFLSSPLKPLSSSGYASHKEKEMVASL-FCKLAALVRHRIS-LFG 2938

V098 VLLPLVDQYFTNHRLYFLSSPLKPLSSSGYASHKEKEMVASL-FCKLAALVRHRIS-LFG 2937

V498 VLLPLVDQYFTNHRLYFLSSPLKPLSSSGYASHKEKEMVASL-FCKLAALVRHRIS-LFG 2917

V497 SDSTTMVSCLHILAQTLDTRTVMKSGSELVKAGLRAFFENAAEDLEKTSENLKLGKFTHS 2997

V672 SDSTTMVSCLHILAQTLDTRTVMKSGSELVKAGLRAFFENAAEDLEKTSENLKLGKFTHS 2978

V366 SDSTTMVSCLHILAQTLDTRTVMKSGSELVKAGLRAFFENAAEDLEKTSENLKLGKFTHS 1125

V993 SDSTTMVSCLHILAQTLDTRTVMKSGSELVKAGLRAFFENAAEDLEKTSENLKLGKFTHS 2997

V671 SDSTTMVSCLHILAQTLDTRTVMKSGSELVKAGLRAFFENAAEDLEKTSENLKLGKFTHS 2998

V098 SDSTTMVSCLHILAQTLDTRTVMKSGSELVKAGLRAFFENAAEDLEKTSENLKLGKFTHS 2997

V498 SDSTTMVSCLHILAQTLDTRTVMKSGSELVKAGLRAFFENAAEDLEKTSENLKLGKFTHS 2977

V497 RTQIKGVSQNINYTTVALLPILTSIFEHIAQHQFGVDLLLSDVQVSCYHILCSLYSLGTG 3057

V672 RTQIKGVSQNINYTTVALLPILTSIFEHIAQHQFGVDLLLSDVQVSCYHILCSLYSLGTG 3038

V366 RTQIKGVSQNINYTTVALLPILTSIFEHIAQHQFGVDLLCNVLLLENVWLP--------- 1166

V993 RTQIKGVSQNINYTTVALLPILTSIFEHIAQHQFGVDLLLSDVQVSCYHILCSLYSLGTG 3057

V671 RTQIKGVSQNINYTTVALLPILTSIFEHIAQHQFGVDLLLSDVQVSCYHILCSLYSLGTG 3058

V098 RTQIKGVSQNINYTTVALLPILTSIFEHIAQHQFGVDLLLSDVQVSCYHILCSLYSLGTG 3057

V498 RTQIKGVSQNINYTTVALLPILTSIFEHIAQHQFGVDLLLSDVQVSCYHILCSLYSLGTG 3037

V497 KNIYVERQRPALGECLASLAAAIPVAFLEPSLNRHNPLSVFNTKTPRERSILGMPDKVED 3117

V672 KNIYVERQRPALGECLASLAAAIPVAFLEPSLNRHNPLSVFNTKTPRERSILGMPDKVED 3098

V993 KNIYVERQRPALGECLASLAAAIPVAFLEPSLNRHNPLSVFNTKTPRERSILGMPDKVED 3117

V671 KNIYVERQRPALGECLASLAAAIPVAFLEPSLNRHNPLSVFNTKTPRERSILGMPDKVED 3118

V098 KNIYVERQRPALGECLASLAAAIPVAFLEPSLNRHNPLSVFNTKTPRERSILGMPDKVED 3117

V498 KNIYVERQRPALGECLASLAAAIPVAFLEPSLNRHNPLSVFNTKTPRERSILGMPDKVED 3097

V497 MCPDIPQLEGLMKEINDLAESGARYTEMPHVIEVILPMLCNYLSYWWERGPENLPPSTGP 3177

V672 MCPDIPQLEGLMKEINDLAESGARYTEMPHVIEVILPMLCNYLSYWWERGPENLPPSTGP 3158

V993 MCPDIPQLEGLMKEINDLAESGARYTEMPHVIEVILPMLCNYLSYWWERGPENLPPSTGP 3177

V671 MCPDIPQLEGLMKEINDLAESGARYTEMPHVIEVILPMLCNYLSYWWERGPENLPPSTGP 3178

V098 MCPDIPQLEGLMKEINDLAESGARYTEMPHVIEVILPMLCNYLSYWWERGPENLPPSTGP 3177

V498 MCPDIPQLEGLMKEINDLAESGARYTEMPHVIEVILPMLCNYLSYWWERGPENLPPSTGP 3157

V497 CCTKVTSEHLSLILGNILKIINNNLGIDEASWMKRIAVYAQPIISKARPDLLRSHFIPTL 3237

V672 CCTKVTSEHLSLILGNILKIINNNLGIDEASWMKRIAVYAQPIISKARPDLLRSHFIPTL 3218

V993 CCTKVTSEHLSLILGNILKIINNNLGIDEASWMKRIAVYAQPIISKARPDLLRSHFIPTL 3237

V671 CCTKVTSEHLSLILGNILKIINNNLGIDEASWMKRIAVYAQPIISKARPDLLRSHFIPTL 3238

V098 CCTKVTSEHLSLILGNILKIINNNLGIDEASWMKRIAVYAQPIISKARPDLLRSHFIPTL 3237

V498 CCTKVTSEHLSLILGNILKIINNNLGIDEASWMKRIAVYAQPIISKARPDLLRSHFIPTL 3217

V497 EKLKKKAVKTVQEEEQLKTDGKGDTQEAELLILDEFAVLCRDLYAFYPMLIRYVDNNRSN 3297

V672 EKLKKKAVKTVQEEEQLKTDGKGDTQEAELLILDEFAVLCRDLYAFYPMLIRYVDNNRSN 3278

V993 EKLKKKAVKTVQEEEQLKTDGKGDTQEAELLILDEFAVLCRDLYAFYPMLIRYVDNNRSN 3297

V671 EKLKKKAVKTVQEEEQLKTDGKGDTQEAELLILDEFAVLCRDLYAFYPMLIRYVDNNRSN 3298

V098 EKLKKKAVKTVQEEEQLKTDGKGDTQEAELLILDEFAVLCRDLYAFYPMLIRYVDNNRSN 3297

V498 EKLKKKAVKTVQEEEQLKTDGKGDTQEAELLILDEFAVLCRDLYAFYPMLIRYVDNNRSN 3277

V497 WLKSPDPDSDQLFRMVAEVFILWCKSHNFKREEQNFVIQNEINNLAFLTGDSKSKMSK-- 3352

V672 WLKSPDPDSDQLFRMVAEVFILWCKSHNFKREEQNFVIQNEINNLAFLTGDSKSKMSKAM 3338

V993 WLKSPDPDSDQLFRMVAEVFILWCKSHNFKREEQNFVIQNEINNLAFLTGDSKSKMSKAM 3357

V671 WLKSPDPDSDQLFRMVAEVFILWCKSHNFKREEQNFVIQNEINNLAFLTGDSKSKMSK-- 3355

V098 WLKSPDPDSDQLFRMVAEVFILWCKSHNFKREEQNFVIQNEINNLAFLTGDSKSKMSKAM 3357

V498 WLKSPDPDSDQLFRMVAEVFILWCKSHNFKREEQNFVIQNEINNLAFLTGDSKSKMSK-- 3334

V497 ---SGGQDQERKKTKRRGDLYSIQTSLIVAALKKMLPIGLNMCTPGDQELISLAKSRYSC 3412

V672 QVKSGGQDQERKKTKRRGDLYSIQTSLIVAALKKMLPIGLNMCTPGDQELISLAKSRYSC 3398

V993 QVKSGGQDQERKKTKRRGDLYSIQTSLIVAALKKMLPIGLNMCTPGDQELISLAKSRYSC 3417

V671 ---SGGQDQERKKTKRRGDLYSIQTSLIVAALKKMLPIGLNMCTPGDQELISLAKSRYSC 3413

V098 QVKSGGQDQERKKTKRRGDLYSIQTSLIVAALKKMLPIGLNMCTPGDQELISLAKSRYSC 3417

V498 ---SGGQDQERKKTKRRGDLYSIQTSLIVAALKKMLPIGLNMCTPGDQELISLAKSRYSC 3392

V497 RDTDEEVKEHLRNNLHLQEKSDDPAVKWQLNLYKDVLRNDEPSNPEKTVERVQSISAALF 3472

V672 RDTDEEVKEHLRNNLHLQEKSDDPAVKWQLNLYKDVLRNDEPSNPEKTVERVQSISAALF 3458

V993 RDTDEEVKEHLRNNLHLQEKSDDPAVKWQLNLYKDVLRNDEPSNPEKTVERVQSISAALF 3477

V671 RDTDEEVKEHLRNNLHLQEKSDDPAVKWQLNLYKDVLRNDEPSNPEKTVERVQSISAALF 3473

V098 RDTDEEVKEHLRNNLHLQEKSDDPAVKWQLNLYKDVLRNDEPSNPEKTVERVQSISAALF 3477

V498 RDTDEEVKEHLRNNLHLQEKSDDPAVKWQLNLYKDVLRNDEPSNPEKTVERVQSISAALF 3452

-------------**CALM**-------------

V497 HLEQVEQPLRSKKAVWHKLLSKQRKRAVVACFRMAPLYNLPRHRS-INLFLHGYQRFWIE 3531

V672 HLEQVEQPLRSKKAVWHKLLSKQRKRAVVACFRMAPLYNLPRNLP-GLKMRKKRRQRGNL 3517

V993 HLEQVEQPLRSKKAVWHKLLSKQRKRAVVACFRMAPLYNLPRNLP-GLKMRKKRRQRGNL 3536

V671 HLEQVEQPLRSKKAVWHKLLSKQRKRAVVACFRMAPLYNLPRNLP-GLKMRKKRRQRGNL 3532

V098 HLEQVEQPLRSKKAVWHKLLSKQRKRAVVACFRMAPLYNLPRHRS-INLFLHGYQRFWIE 3536

V498 HLEQVEQPLRSKKAVWHKLLSKQRKRAVVACFRMAPLYNLPRHKINNFFLITFQRVWLEK 3512

V497 TEAHFFEEKLVQDLAKSPRVEDEEEEETERQPDPLHQIILHFSRNALTERSKLEDDPLYT 3591

V672 THFTRSFCILVATRSRRGANWKMIHCTLPIPA---------------------------- 3549

V993 THFTRSFCILVATRSRRGANWKMIHCTLPIPA---------------------------- 3568

V671 THFTRSFCILVATRSRRGANWKMIHCTLPIPA---------------------------- 3564

V098 TEAHFFEEKLVQDLAKSPRVEDEEEEETERQPDPLHQIILHFSRNALTERSKLEDDPLYT 3596

V498 VNEKTQYDRLIPILMKSPRVEDEEEEETERQPDPLHQIILHFSRNALTERSKLEDDPLYT 3572

V497 SYSSMMAKSCQSGEDEEEEEDKEKTFEEKEMEKQKTLYQQARLHERGAAEMVLQMISASK 3651

V098 SYSSMMAKSCQSGEDEEEEEDKEKTFEEKEMEKQKTLYQQARLHERGAAEMVLQMISASK 3656

V498 SYSSMMAKSCQSGEDEEEEEDKEKTFEEKEMEKQKTLYQQARLHERGAAEMVLQMISASK 3632

V497 GEMSPMVVETLKLGIAILNGGNAGVQQKMLDYLKEKKDAGFFQSLSGLMQSCSVLDLNAF 3711

V098 GEMSPMVVETLKLGIAILNGGNAGVQQKMLDYLKEKKDAGFFQSLSGLMQSCSVLDLNAF 3716

V498 GEMSPMVVETLKLGIAILNGGNAGVQQKMLDYLKEKKDAGFFQSLSGLMQSCSVLDLNAF 3692

V497 ERQNKAEGLGMVTEEGTLIVRERGEKVLQNDEFTQDLFRFLQLLCEGHNSDFQNFLRTQM 3771

V098 ERQNKAEGLGMVTEEGTLIVRERGEKVLQNDEFTQDLFRFLQLLCEGHNSDFQNFLRTQM 3776

V498 ERQNKAEGLGMVTEEGT------REKVLQNDEFTQDLFRFLQLLCEGHNSDFQNFLRTQM 3746

V497 GNTTTVNIIISTVDYLLRLQESISDFYWYYSGKDIIDESGQHNFSKALAVTKQIFNSLTE 3831

V098 GNTTTVNIIISTVDYLLRLQESISDFYWYYSGKDIIDESGQHNFSKALAVTKQIFNSLTE 3836

V498 GNTTTVNIIISTVDYLLRLQESISDFYWYYSGKDIIDESGQHNFSKALAVTKQIFNSLTE 3806

V497 YIQGPCIGNQQSLAHSRLWDAVVGFLHVFANMQMKLSQDSSQIELLKELLDLLQDMVVML 3891

V098 YIQGPCIGNQQSLAHSRLWDAVVGFLHVFANMQMKLSQDSSQIELLKELLDLLQDMVVML 3896

V498 YIQGPCIGNQQSLAHSRLWDAVVGFLHVFANMQMKLSQDSSQIELLKELLDLLQDMVVML 3866

**Ca^2+^**

V497 LSLLEGWAVWYN------------------------------------------------ 3903

V098 LSLLEGNVVNGTIGKQMVDTLVESSTNVEMILKFFDMFLKLKDLTSSDTFKEYDPDGKGI 3956

V498 LSLLEGNVVNGTIGKQMVDTLVESSTNVEMILKFFDMFLKLKDLTSSDTFKEYDPDGKGI 3926

V589 ----------------MVDTLVESSTNVEMILKFFDMFLKLKDLTSSDTFKEYDPDGKGI 44

V714 ----------------MVDTLVESSTNVEMILKFFDMFLKLKDLTSSDTFKEYDPDGKGI 44

V757 ----------------MVDTLVESSTNVEMILKFFDMFLKLKDLTSSDTFKEYDPDGKGI 44

V098 ISRKEFQKAMEGLKQYTQSEIDFLLSCTEADENDMFNYVDFVERFHEPAKDIGFNVAVLL 4016

V498 ISRKEFQKAMEGLKQYTQSEIDFLLSCTEADENDMFNYVDFVERFHEPAKDIGFNVAVLL 3986

V589 ISRKEFQKAMEGLKQYTQSEIDFLLSCTEADENDMFNYVDFVERFHEPAKDIGFNVAVLL 104

V714 ISRKEFQKAMEGLKQYTQSEIDFLLSCTEADENDMFNYVDFVERFHEPAKDIGFNVAVLL 104

V757 ISRKEFQKAMEGLKQYTQSEIDFLLSCTEADENDMFNYVDFVERFHEPAKDIGFNVAVLL 104

V098 TNLSEHMPNDSRLKSLLDPAESVLNYFEPYLGRIEIMGGAKKIERVYFEISESSRTQWEK 4076

V498 TNLSEHMPNDSRLKSLLDPAESVLNYFEPYLGRIEIMGGAKKIERVYFEISESSRTQWEK 4046

V589 TNLSEHMPNDSRLKSLLDPAESVLNYFEPYLGRIEIMGGAKKIERVYFEISESSRTQWEK 164

V714 TNLSEHMPNDSRLKSLLDPAESVLNYFEPYLGRIEIMGGAKKIERVYFEISESSRTQWEK 164

V757 TNLSEHMPNDSRLKSLLDPAESVLNYFEPYLGRIEIMGGAKKIERVYFEISESSRTQWEK 164

V098 PQVKESKRQFIFDVVNEGGEQEKMELFVNFCEDTIFEMQLASQISESDSTDRPEEEEEED 4136

V498 PQVKESKRQFIFDVVNEGGEQEKMELFVNFCEDTIFEMQLASQISESDSTDRPEEEEEED 4106

V589 PQVKESKRQFIFDVVNEGGEQEKMELFVNFCEDTIFEMQLASQISESDSTDRPEEEEEED 224

V714 PQVKESKRQFIFDVVNEGGEQEKMELFVNFCEDTIFEMQLASQISESDSTDRPEEEEEED 224

V757 PQVKESKRQFIFDVVNEGGEQEKMELFVNFCEDTIFEMQLASQISESDSTDRPEEEEEED 224

V098 EDSAYSIETEGEEEEKSFESASAFTMACVSVKRNVTKFLKRATLKNLRKQYRNVKKMSAK 4196

V498 EDSAYSIETEGEEEEKSFESASAFTMACVSVKRNVTKFLKRATLKNLRKQYRNVKKMSAK 4166

V589 EDSAYSIETEGEEEEKSFESASAFTMACVSVKRNVTKFLKRATLKNLRKQYRNVKKMSAK 284

V714 EDSAYSIETEGEEEEKSFESASAFTMACVSVKRNVTKFLKRATLKNLRKQYRNVKKMSAK 284

V757 EDSAYSIETEGEEEEKSFESASAFTMACVSVKRNVTKFLKRATLKNLRKQYRNVKKMSAK 284

---------**TM1**---------

V098 ELVKVFFSFFWMLFVGLFQLLFTIFGGIFQILWNTVFGGGLVEGAKNIRVTKILGDMPDP 4256

V498 ELVKVFFSFFWMLFVGLFQLLFTIFGGIFQILWNTVFGGGLVEGAKNIRVTKILGDMPDP 4226

V589 ELVKVFFSFFWMLFVGLFQLLFTIFGGIFQILWNTVFGGGLVEGAKNIRVTKILGDMPDP 344

V714 ELVKVFFSFFWMLFVGLFQLLFTIFGGIFQILWNTVFGGGLVEGAKNIRVTKILGDMPDP 344

V757 ELVKVFFSFFWMLFVGLFQLLFTIFGGIFQILWNTVFGGGLVEGAKNIRVTKILGDMPDP 344

V098 TQFGIHDDVIETDRAEVTEPGVTTELVHFVKGEAGDTDIMSDLFGIHSKKEGGLKQGPEV 4316

V498 TQFGIHDDVIETDRAEVTEPGVTTELVHFVKGEAGDTDIMSDLFGIHSKKEGGLKQGPEV 4286

V589 TQFGIHDDVIETDRAEVTEPGVTTELVHFVKGEAGDTDIMSDLFGIHSKKEGGLKQGPEV 404

V714 TQFGIHDDVIETDRAEVTEPGVTTELVHFVKGEAGDTDIMSDLFGIHSKKEGGLKQGPEV 404

V757 TQFGIHDDVIETDRAEVTEPGVTTELVHFVKGEAGDTDIMSDLFGIHSKKEGGLKQGPEV 404

V098 GLGDLSEIIGKDEPPTLESTVRKKRKAQAAEMKAVHEAEGKAESEKAESLKNL------- 4369

V498 GLGDLSEIIGKDEPPTLESTVRKKRKAQAAEMKAVHEAEGKAESEKADMEDREKEDKIKE 4346

V589 GLGDLSEIIGKDEPPTLESTVRKKRKAQAAEMKAVHEAEGKAESEKADMEDREKEDKIKE 464

V714 GLGDLSEIIGKDEPPTLESTVRKKRKAQAAEMKAVHEAEGKAESEKADMEDREKEDKIKE 464

V757 GLGDLSEIIGKDEPPTLESTVRKKRKAQAAEMKAVHEAEGKAESEKADMEDREKEDKIKE 464

---------

V498 EGQTDYLWADVTVKKTRRRGQKAEKPEAFMANFFKGLEIYQTKLLHYLARNFYNLRFLAL 4406

V589 EGQTDYLWADVTVKKTRRRGQKAEKPEAFMANFFKGLEIYQTKLLHYLARNFYNLRFLAL 524

V714 EGQTDYLWADVTVKKTRRRGQKAEKPEAFMANFFKGLEIYQTKLLHYLARNFYNLRFLAL 524

V757 EGQTDYLWADVTVKKTRRRGQKAEKPEAFMANFFKGLEIYQTKLL--------------- 509

**TM2**---------

V498 FVAFAINFILLFYKVTEEPLEEETEDVANLWNSFNDDDEEEAMVFFVLQESTGYMAPTLR 4466

V589 FVAFAINFILLFYKVTEEPLEEETEDVANLWNSFNDDDEEEAMVFFVLQESTGYMAPTLR 584

V714 FVAFAINFILLFYKVTEEPLEEETEDVANLWNSFNDDDEEEAMVFFVLQESTGYMAPTLR 584

V757 --------------VTEEPLEEETEDVANLWNSFNDDDEEEAMVFFVLQESTGYMAPTLR 555

---------**TM3**---------

V498 ALAIVHTIISLVCVVGYYCLKVPLVVFKREKEIARKLEFDGLYITEQPSEDDIKGQWDRL 4526

V589 ALAIVHTIISLVCVVGYYCLKVPLVVFKREKEIARKLEFDGLYITEQPSEDDIKGQWDRL 644

V714 ALAIVHTIISLVCVVGYYCLKVPLVVFKREKEIARKLEFDGLYITEQPSEDDIKGQWDRL 644

V757 ALAIVHTIISLVCVVGYYCLKVPLVVFKREKEIARKLEFDGLYITEQPSEDDIKGQWDRL 615

V498 VINTPSFPNNYWDKFVKRKVINKYGDLYGAERIAELLGLDKNALDFSPVEEAKAEAASLV 4586

V589 VINTPSFPNNYWDKFVKRKVINKYGDLYGAERIAELLGLDKNALDFSPVEEAKAEAASLV 704

V714 VINTPSFPNNYWDKFVKRKVINKYGDLYGAERIAELLGLDKNALDFSPVEEAKAEAASLV 704

V757 VINTPSFPNNYWDKFVKRKVINKYGDLYGAERIAELLGLDKNALDFSPVEEAKAEAASLV 675

---------**TM4**--------- ---------**TM5**---------

V498 SWLSSIDMKYHIWKLGVVFTDNSFLYLAWYTTMSVLGHYNNFFFAAHLLDIAMGFKTLRT 4646

V589 SWLSSIDMKYHIWKLGVVFTDNSFLYLAWYTTMSVLGHYNNFFFAAHLLDIAMGFKTLRT 764

V714 SWLSSIDMKYHIWKLGVVFTDNSFLYLAWYTTMSVLGHYNNFFFAAHLLDIAMGFKTLRT 764

V757 SWLSSIDMKYHIWKLGVVFTDNSFLYLAWYTTMSVLGHYNNFFFAAHLLDIAMGFKTLRT 735

---------**TM6**---------

V498 ILSSVTHNGKQLVLTVGLLAVVVYLYTVVAFNFFRKFYNKSEDDDEPDMKCDDMMTCYLF 4706

V589 ILSSVTHNGKQLVLTVGLLAVVVYLYTVVAFNFFRKFYNKSEDDDEPDMKCDDMMTCYLF 824

V714 ILSSVTHNGKQLVLTVGLLAVVVYLYTVVAFNFFRKFYNKSEDDDEPDMKCDDMMTCYLF 824

V757 ILSSVTHNGKQLVLTVGLLAVVVYLYTVVAFNFFRKFYNKSEDDDEPDMKCDDMMTCYLF 795

---**Pore**--- ---------**TM7**---------

V498 HMYVGVRAGGGIGDEIEDPAGDPYEMYRIVFDITFFFFVIVILLAIIQGLIIDAFGELRD 4766

V589 HMYVGVRAGGGIGDEIEDPAGDPYEMYRIVFDITFFFFVIVILLAIIQGLIIDAFGELRD 884

V714 HMYVGVRAGGGIGDEIEDPAGDPYEMYRIVFDITFFFFVIVILLAIIQGLIIDAFGELRD 884

V096 -MYVGVRAGGGIGDEIEDPAGDPYEMYRIVFDITFFFFVIVILLAIIQGLIIDAFGELRD 59

V757 HMYVGVRAGGGIGDEIEDPAGDPYEMYRIVFDITFFFFVIVILLAIIQGLIIDAFGELRD 855

V498 QQEQVREDMETKCFICGIGNDYFDTTPHGFETHTLQEHNLANYLFFLMYLINKDETEHTG 4826

V589 QQEQVREDMETKCFICGIGNDYFDTTPHGFETHTLQEHNLANYLFFLMYLINKDETEHTG 944

V714 QQEQVREDMETKCFICGIGNDYFDTTPHGFETHTLQEHNLANYLFFLMYLINKDETEHTG 944

V096 QQEQVREDMETKCFICGIGNDYFDTTPHGFETHTLQEHNLANYLFFLMYLINKDETEHTG 119

V757 QQEQVREDMETKCFICGIGNDYFDTTPHGFETHTLQEHNLANYLFFLMYLINKDETEHTG 915

V498 QESYVWKMYQERCWDFFPAGDCFRKQYEDQLG 4858

V589 QESYVWKMYQERCWDFFPAGDCFRKQYEDQLG 976

V714 QESYVWKMYQERCWDFFPAGDCFRKQYEDQLG 976

V096 QESYVWKMYQERCWDFFPAGDCFRKQYEDQLG 151

V757 QESYVWKMYQERCWDFFPAGDCFRKQYEDQLG 947
