# Supplementary material for: Smooth Muscle Cell Genome Browser: Enabling the Identification of Novel Serum Response Factor Target Genes
Source: PLoS One. 2015 Aug 4;10(8):e0133751. doi: 10.1371/journal.pone.0133751 (PMC4524680; doi:10.1371/journal.pone.0133751)
Supplement: S10 Table — (DOC) [file pone.0133751.s019.doc]

**S10 Table. Sequence permutations of CArG Boxes in the CArGome.**

| /(CC[AT][AT][AT][AT][AT][AT]GG)/ |
| --- |
| /([ATG]C[AT][AT][AT][AT][AT][AT]GG)/ |
| /(C[ATG][AT][AT][AT][AT][AT][AT]GG)/ |
| /(CC[CG][AT][AT][AT][AT][AT]GG)/ |
| /(CC[AT][CG][AT][AT][AT][AT]GG)/ |
| /(CC[AT][AT][CG][AT][AT][AT]GG)/ |
| /(CC[AT][AT][AT][CG][AT][AT]GG)/ |
| /(CC[AT][AT][AT][AT][CG][AT]GG)/ |
| /(CC[AT][AT][AT][AT][AT][CG]GG)/ |
| /(CC[AT][AT][AT][AT][AT][AT][ATC]G)/ |
| /(CC[AT][AT][AT][AT][AT][AT]G[ATC])/ |
